# Research Progress of Fluorescent Probes for Detection of Glutathione (GSH): Fluorophore, Photophysical Properties, Biological Applications

**DOI:** 10.3390/molecules29184333

**Published:** 2024-09-12

**Authors:** Yao Wang, Yanfei Li, Jinbo Cao, Xiyan Yang, Jiaxiang Huang, Mingyue Huang, Shaobin Gu

**Affiliations:** 1 Henan International Joint Laboratory of Food Green Processing and Quality Safety Control, College of Food and Bioengineering, Henan University of Science and Technology, Luoyang 471023, China; 220320070754@stu.haust.edu.cn (Y.L.); 2023010186@m.scnu.edu.cn (J.C.); 220320070681@stu.haust.edu.cn (X.Y.); 220320070733@stu.haust.edu.cn (J.H.); 230320070742@stu.haust.edu.cn (M.H.); 2 Guangzhou Key Laboratory of Analytical Chemistry for Biomedicine, School of Chemistry, South China Normal University, Guangzhou 511400, China

**Keywords:** glutathione, fluorescent probe, fluorophore, living cell imaging

## Abstract

Intracellular biothiols, including cysteine (Cys), glutathione (GSH), and homocysteine (Hcy), play a critical role in many physiological and pathological processes. Among them, GSH is the most abundant non-protein mercaptan (1–10 mM) in cells, and the change in GSH concentration level is closely related to the occurrence of many diseases, such as Parkinson’s disease, Alzheimer’s disease, and neurological diseases. Fluorescent probes have attracted much attention due to their advantages of high specificity, high sensitivity, high selectivity, low cost, and high quantum yield. Methods that use optical probes for selective detection of GSH in vitro and in vivo are in high demand. In this paper, we reviewed the most recent five years of research on fluorescence probes for the detection of GSH, including the specific detection of GSH, dual-channel identification of GSH and other substances, and the detection of GSH and other biothiols. According to the type of fluorophore, we classified GSH fluorescent probes into eight classes, including BODIPY, 1,8-Naphthalimide, coumarin, xanthene, rhodamine, cyanine, benzothiazoles, and others. In addition, we roundly discuss the synthesis, detection mechanism, photophysical properties, and biological applications of fluorescent probes. We hope that this review will inspire the exploration of new fluorescent probes for GSH and other related analyses.

## 1. Introduction

**Figure 1:**
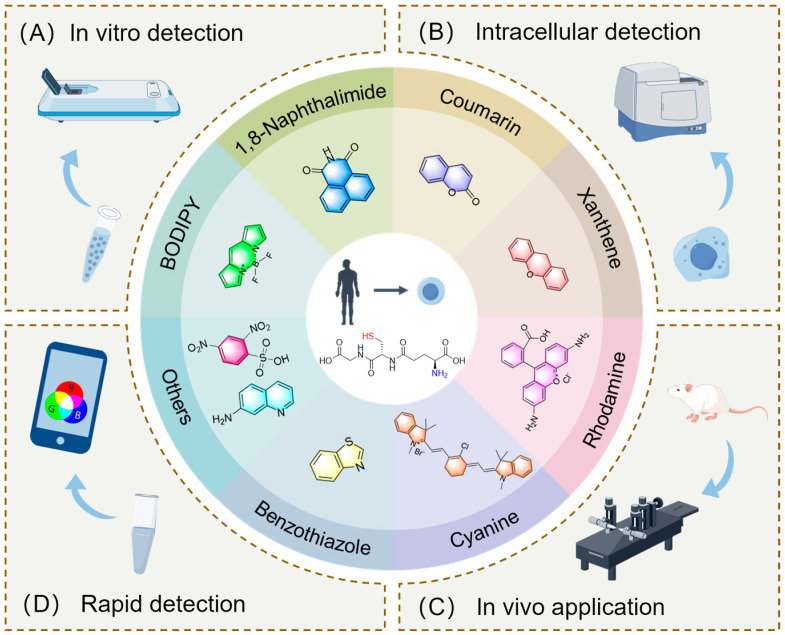
Summary of GSH probes based on different fluorophores and applications.

Intracellular biothiols, including cysteine (Cys), glutathione (GSH), and homocysteine (Hcy) (Figure 1), play a critical role in many physiological and pathological processes [1,2]. GSH is composed of glutamic acid, cysteine, and glycin [3]. It is widely present in living cells at intracellular concentrations of 1–10 mM, much higher than Cys (30–200 μM) and Hcy (5–15 μM) [4,5]. It plays a vital role in biochemical defense systems and has a range of physiological functions [6]. As an important antioxidant in the body, it protects the sulfhydryl groups in liver proteins and enzymes, maintaining their REDOX state through the mutual conversion of the forms of reduced sulfhydryl (GSH) and disulfide oxide (GSSG) [7,8]. GSH levels vary widely between healthy and unhealthy people, and abnormal GSH levels in tissues have been linked to a variety of diseases, such as liver damage [9], heart disease [10], leukopenia [11], AIDS [12], Parkinson’s disease [13], Alzheimer’s disease [14], and many neurological disorders [15,16,17]. Therefore, it is of great significance to establish a high specificity and high sensitivity GSH detection method for clinical diagnosis and basic research.

At present, GSH detection methods mainly include the enzyme cycle method [18,19], high-performance liquid chromatography (HPLC) [20,21,22], liquid chromatography/mass spectrometry (LC/MS) [23,24], colorimetry [25,26,27], flow cytometry (FCM) [28,29,30], capillary electrophoresis [31,32], the electrochemical method [33,34], electrochemiluminescence (ECL) [35,36], and the nuclear magnetic resonance method (NMR) [37,38]. However, fluorescent probes have attracted wide attention due to their advantages of high specificity, high sensitivity, high selectivity, low cost, and high quantum yield. What’s more, GSH in vivo and in vitro can be visually detected using fluorescent probes [39,40]. At present, remarkable progress has been made in the development and application of GSH probes. In this paper, we review the fluorescent probes used to detect GSH in the last five years and classify them according to fluorophore type. As shown in Figure 1, it is divided into eight categories: BODIPY, 1,8-Naphthalimide, coumarin, xanthene, rhodamine, cyanine, benzothiazoles, and others.

## 2. Fluorescent Probe of GSH

**Figure 2:**
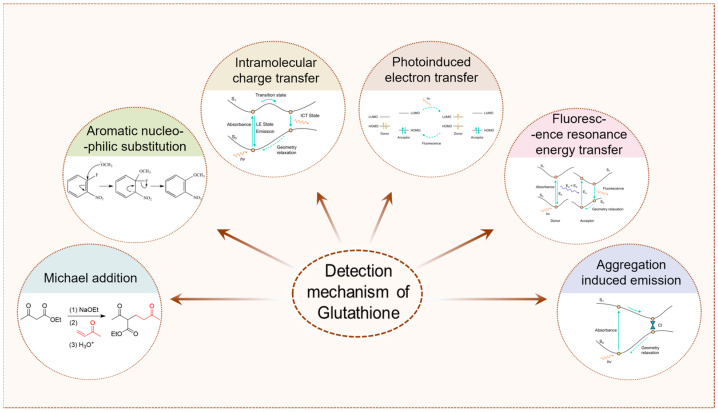
Summary of the detection mechanism of GSH. The detection mechanism schematic diagram of intramolecular charge transfer, photoinduced electron transfer, fluorescence resonance energy transfer, and aggregation-induced emission are reproduced from the website https://mancekou.github.io/posts/3a0936ab/ (accessed on 18 August 2024).

The recent five years of GSH fluorescent probes are reviewed in eight sections, each of which is classified according to fluorophore type. We discuss the synthesis of probes, detection mechanism, photophysical properties, selectivity, sensitivity, and exogenous and endogenous GSH recognition. As shown in Figure 2, the detection mechanisms of GSH in this paper include Michael addition, aromatic nucleophilic substitution (S_N_Ar), intramolecular charge transfer (ICT), photoinduced electron transfer (PET), fluorescence resonance energy transfer (FRET), and aggregation-induced emission (AIE). In each of the following sections, we collate GSH-specific assays, dual-channel probes for identifying GSH and other substances, and multi-channel probes for identifying GSH and other mercaptans. We hope that our review can provide a useful reference for more researchers to develop the next generation of fluorescent probes for detecting GSH.

### 2.1. Fluorescent Probe Based on BODIPY

**Figure 3:**
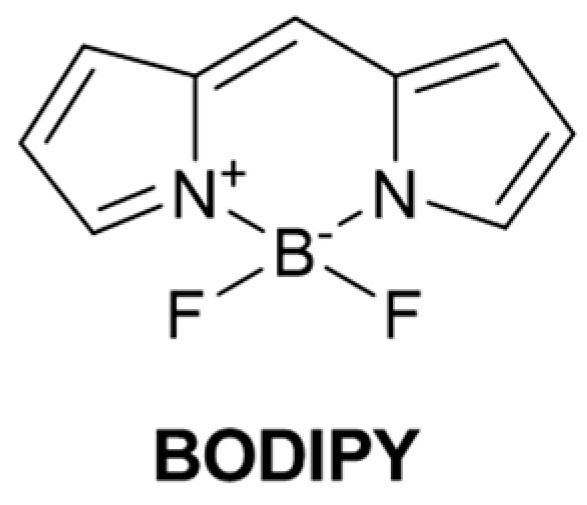
Structure of fluorophore BODIPY.

BODIPY fluorophore is composed of two pyrrole rings and a boron atom connected by a benzene ring or other substituent group [41] (Figure 3). BODIPY has the advantages of structural versatility, unique small Stokes shift, high fluorescence quantum yield, high molar extinction coefficient, high solubility, good optical stability, and pH insensitivity and is widely used in biomarkers, fluorescence probes, and biological imaging [42].

**Figure 4:**
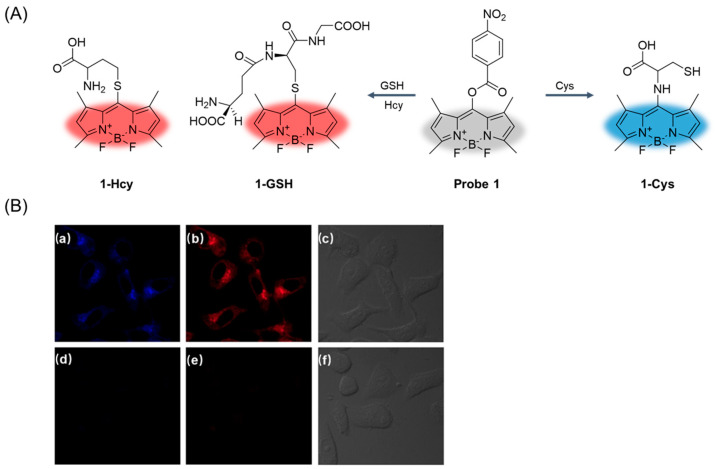
(**A**) Structure of **Probe 1** and its reaction with Cys and Hcy/GSH. (**B**) Confocal fluorescence and bright-field images of living HeLa cells incubated with **Probe 1** (10 μM) for 20 min: (**a**) blue channel at 460–500 nm, (**b**) red channel at 570–620 nm, (**c**) bright-field transmission image; confocal fluorescence and bright-field images of living HeLa cells incubated with the **Probe 1** (10 μM) for 20 min after pre-incubation with 5 mM NEM for 30 min: (**d**) blue channel at 460–500 nm, (**e**) red channel at 570–620 nm, (**f**) bright-field transmission image. Reproduced with permission from ref. [43]. Copyright 2019 Elsevier.

In 2018, Ji et al. [43] reported a fluorescent probe, **Probe 1**, for simultaneous differentiation of Cys and Hcy/GSH from dual emission channels (Figure 4A). BODIPY dye was used as the fluorophore of the probe, 1-, 3-, 5-, and 7-positions contained tetramethyl groups, and the median position contained N-, O-, or S-aryl substituents as leaving groups. a-PET or d-PET effect occurred during the recognition process. When excited at 420 nm, the fluorescence of Cys was enhanced at 439 nm, when excited at 500 nm, the fluorescence of Hcy/GSH was enhanced at 544 nm, and the fluorescence intensity increased with time. The fluorescence response of the probe to Cys and Hcy/GSH was similar. The fluorescence enhancement increased with the increase in pH value, and the optimal pH range was 7.2–7.6. The probe has the advantages of high selectivity and sensitivity. The probe can simultaneously sense Cys and Hcy/GSH in HeLa cells using multicolor imaging (Figure 4B) and provides a suitable strategy for multi-component analysis in living systems.

**Figure 5:**
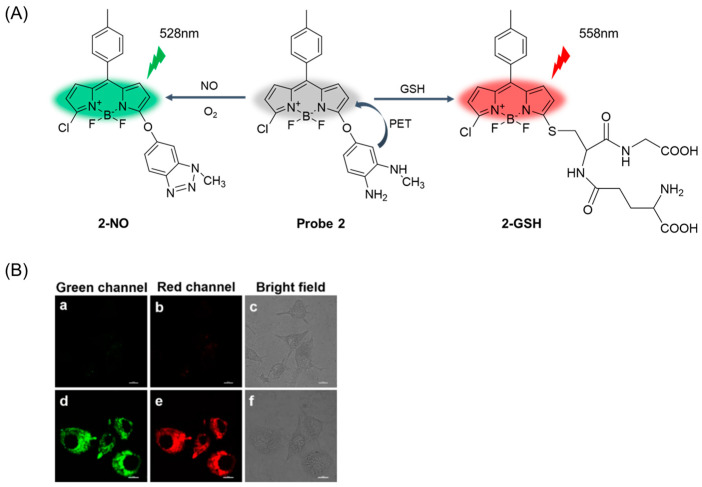
(**A**) Structure of **Probe 2** and its reaction with GSH and NO. (**B**) Endogenous NO and GSH detection in RAW 264.7 cells stained by **Probe 2** (2 μM, 10 min) without (**a**–**c**) and with (**d**–**f**) the stimulation of 20 μg/mL LPS, 50 μg/mL L-Arg, 0.01 μg/mL IFN-γ for 12 h. Emission was collected at 500–550 nm for green channel (excited at 487 nm) and at 550–600 nm for red channel (excited at 487 nm). Scale bar: 10 μm. Reproduced with permission from ref. [44]. Copyright 2019 American Chemical Society.

In 2019, Chen et al. [44] reported a fluorescent probe, **Probe 2**, with BODIPY as a fluorophore and 4-amino-3-(methylamino-phenol) as a responsive fluorescence regulator of nitric oxide (NO) and GSH. As shown in Figure 5A, the probe can detect NO and GSH simultaneously from dual emission channels. A PET effect occurred during recognition, with the NO-mediated conversion of diamine to triazole triggering the fluorescence of the green channel and the GSH-induced aromatic nuclear substitution reaction (S_N_Ar) leading to the red-shifted emission of the red channel. The probe had high selectivity for NO and GSH from different emission channels. The probe was successfully used to detect exogenous and endogenous NO and GSH in macrophages. Importantly, the probe visualized the elevation of NO and GSH in the inflammatory mediators induced by interferon-γ (IFN-γ), lipopolysaccharide (LPS), and L-arginine (L-Arg) in Raw264.7 cells (Figure 5B), thus providing more insight into the physiological relationship between NO and GSH.

**Figure 6:**
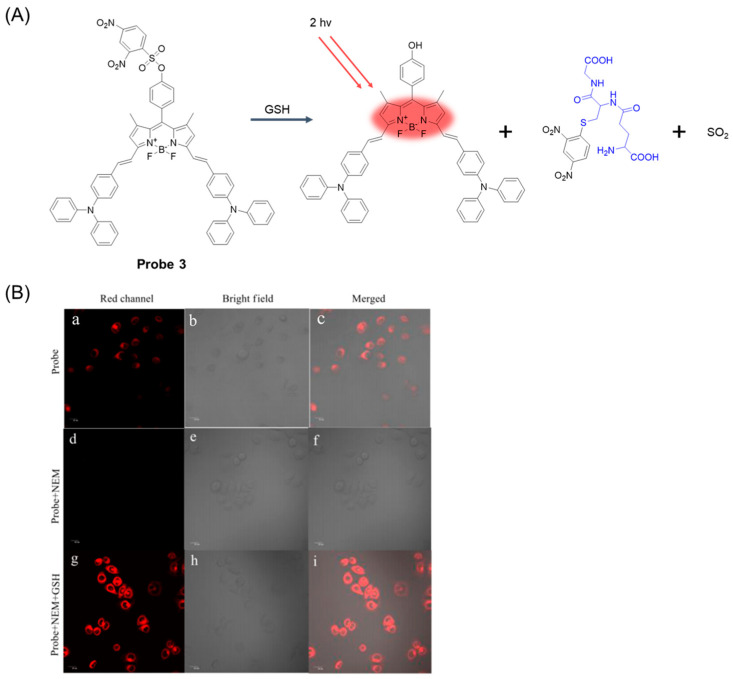
(**A**) Structure of **Probe 3** and its reaction with GSH. (**B**) The top row (**a**–**c**): confocal microscopy images of MCF-7 cells incubated with **Probe 3** (10 μΜ) for 90 min; the middle (**d**–**f**): confocal microscopy images of MCF-7 cells preincubated with NEM (0.5 mM) for 30 min and then treated with **Probe 3** (10 μΜ) for 90 min; the bottom row (**g**–**i**): confocal microscopy images of MCF-7 cells incubated with NEM (0.5 mM) for 30 min, then incubated with **Probe 3** (10 μΜ) for 90 min, and finally with the addition of GSH (1 mM). Excitation at 600 nm. Reproduced with permission from ref. [45]. Copyright 2019 Elsevier.

In 2019, Huang et al. [45] developed a near-infrared two-photon fluorescence probe, **Probe 3**, (Figure 6A), which can specifically detect GSH. The probe was conjugated by 4-(diphenylamino) benzaldehyde at the 3- and 5-positions of BODIPY by Knoevenagel condensation as a near-infrared fluorophore with a longer wavelength. 2, 4-dinitrobenzene sulfonyl group (DNBS) was introduced in the 8-position of BODIPY, which acts as a fluorescence quencher and thiol recognition part. The fluorescence intensity at 719 nm increased with the increase in GSH concentration in the probe solution, and the detection limit was 25.46 nM. The intensity of GSH emission from the probe remained constant between pH 6.0 and 9.0. The probe has the advantages of high selectivity, high sensitivity, and low toxicity. In addition, the probe has been successfully used to monitor exogenous and endogenous GSH in MCF-7 cells (Figure 6B) and can be used for cell imaging after the addition of GSH to living cells.

**Figure 7:**
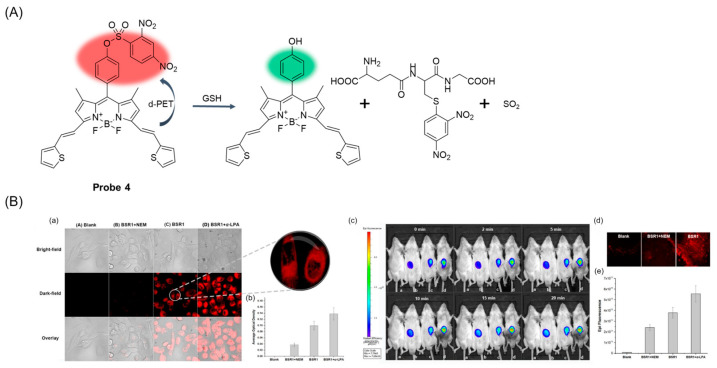
(**A**) Structure of **Probe 4** and its reaction with GSH. (**B**) (**a**) Confocal microscope images of NCI-H1975 cells treated with **BSR1 (Probe 4)** (10 μM, 1 h, λ_ex_ = 633 nm) in presence/absence of NEM or α-thioctic acid. (**b**) Semiquantitative calculation of average fluorescence intensity in cells incubated with **BSR1** conducted by ImageJ software (ImageJ). (**c**) Imaging of endogenous GSH in Kunming mice that were a: blank; b: pretreated with NEM and then injected with **BSR1**; c: treated with **BSR1** only; d: pretreated with α-thioctic acid before being injected with **BSR1**. (**d**) Fluorescence images of **BSR1** in normal liver tissue. (**e**) Semiquantitative analysis of fluorescence intensity in mice of (**c**). Reproduced with permission from ref. [46]. Copyright 2019 American Chemical Society.

In 2019, Chen et al. [46] reported a novel thiophene-vinyl BODIPY near-infrared fluorescent probe, **Probe 4**, which can detect GSH well both in vivo and in vitro. As shown in Figure 7A, BODIPY acts as the fluorophore of the probe, and a d-PET effect occurs during the recognition process. When the concentration of GSH increased gradually, the fluorescence intensity at 663 nm increased gradually, and the detection limit was 83 nM. The probe showed remarkable fluorescence performance for GSH detection at a physiological pH between 6.0 and 9.0. In addition, the probe has the advantages of high selectivity and low toxicity and has shown excellent performance for in vitro and in vivo detection of GSH (Figure 7B). More importantly, the probe can be used for visual monitoring of drug-induced acute liver injury.

**Figure 8:**
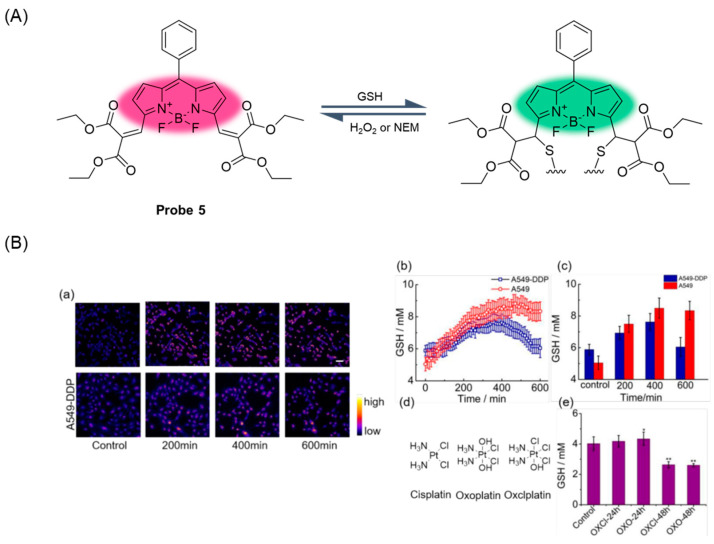
(**A**) Structure of **Probe 5** and its reaction with GSH. (**B**) Real-time variations in intracellular GSH levels in response to divalent platinum (Pt^II^) and tetravalent platinum (Pt^IV^) anticancer drugs. (**a**) Real-time ratiometric imaging of GSH in different timepoints. (**b**) Real-time average values of GSH concentration detected with **Probe 5** (10 μM) in A549 and A549-DDP cells upon cisplatin (10 µM) treatment (A549: n = 77; A549-DDP: n = 86). (**c**) Average GSH concentration detected with **Probe 5** (10 μM) in A549/A549-DDP cells upon cisplatin (10 μM) treatment in different timepoints (A549: n = 77; A549-DDP: n = 86). (**d**) Chemical structures of cisplatin, oxoplatin, and oxclplatin. (**e**) Average values of GSH concentration with **Probe 5** (10 μM) in A549 cells upon oxoplatin and oxclplatin (10 μM) treatment at different timepoints (n = 15). Scale bar: 50 µm. Error bars represent mean ± SD. * *p* < 0.05, ** *p* < 0.01. Reproduced with permission from ref. [47]. Copyright 2020 Creative Commons.

In 2020, Liu et al. [47] reported an example of a rate-fluorescence probe, **Probe 5**, for real-time monitoring of fluctuations in GSH levels in cells treated with cisplatin. As shown in Figure 8A, the probe uses BODIPY as a fluorophore and reacts as a reversible Michael addition with H_2_O_2_ and N-Ethylmaleimide (NEM). The probe was prepared via a conventional TFA-catalyzed condensation reaction of benzaldehyde with pyrrole, followed by oxidation with 2,3-dichloro-5,6-dicyano-1,4benzoquinone (DDQ) and cyclization with BF_3_·Et_2_O in a one-pot three-step process. The probe has the advantages of a wide response range, fast response speed, high selectivity, and low toxicity. In cisplatin-sensitive A549 cells, GSH concentration increased until cell death, while in cisplatin-resistant cell lines, GSH level first increased to the maximum and then fell back to the initial concentration without significant apoptosis (Figure 8B). The results showed that the different trends of GSH fluctuation could help to distinguish cisplatin-resistant cells from cisplatin-sensitive cells.

**Figure 9:**
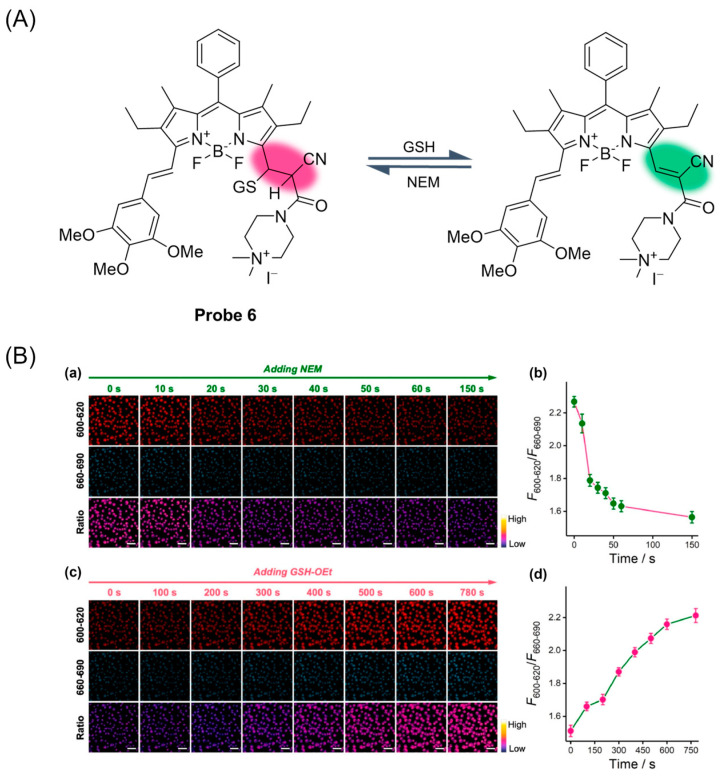
(**A**) Structure of **Probe 6** and its reaction with GSH. (**B**) (**a**,**c**) Real-time fluorescence imaging of GSH in A549 cells upon sequential addition of NEM (2 mM) (**a**) and GSH ethyl ester (GSH-OEt; 5 mM) (**c**) using **Probe 6 (^α^BD-GSH)** (5 µM). Ch_600–620_: Ex/Em: 594/600–620 nm; Ch_660–690_: Ex/Em: 594/660–690 nm; Ratio: Ch_600–620_/Ch_660–690_. Scale bar: 50 μm. (**b**,**d**) Time-dependent changes in the ratio (F_600–620_/F_660–690_) of the two channels upon the addition of NEM (**b**) and then GSH-OEt (**d**) quantified from the respective CLSM images. Error bars represent mean ± SD (n = 3). Reproduced with permission from ref. [48]. Copyright 2021 Elsevier.

In 2020, Zhang et al. [48] reported a case of a reversible fluorescent probe, **Probe 6**, based on BODIPY’s α-arylene Michael addition reaction that could rapidly react with GSH (Figure 9A). The reversible reaction between the probe and GSH was proven by the GSH/NEM cycle test. With the increase in GSH concentration, the fluorescence intensity increased and showed the highest peak at 609 nm. The dissociation constant of the probe was calculated to be 1.55 mM, the fluorescence quantum yield was measured to be 0.11, and the limit of detection was calculated to be 3.4 μM. The probe is not affected by pH in the range of 3.0–10.0, and it has good photostability and high selectivity. The low cytotoxicity of the probe was also demonstrated by methyl thiazolyl tetrazolium (MTT) assay. In addition, the probe has been preliminarily applied to real-time visual detection of GSH dynamics in living cells (Figure 9B).

**Figure 10:**
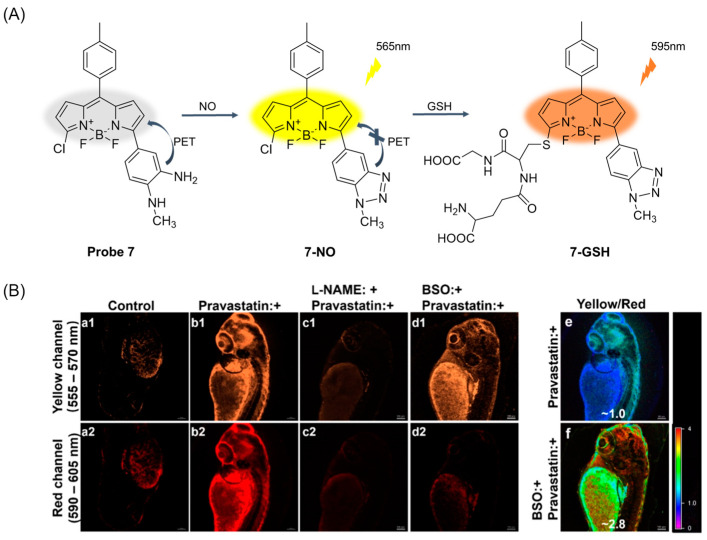
(**A**) Structure of **Probe 7** and its reaction with NO and GSH. (**B**) Fluorescence imaging of living zebrafish treated with **Probe 7** (2 μM, 30 min): (**a1**,**a2**) control image, (**b1**,**b2**) pretreated with pravastatin (50 μM, 24 h), (**c1**,**c2**) pretreated with pravastatin (50 μM, 24 h) and further incubated with _L_-NAME (100 μM, 12 h), and (**d1**,**d2**) pretreated with pravastatin (50 μM, 24 h) and further incubated with BSO (200 μM, 12 h). Emission was collected at 555–570 nm for the yellow channel and at 590–605 nm for the red channel (excited at 487 nm). Ratiometric confocal fluorescence images (yellow/red channels): (**e**) zebrafish treated with pravastatin (50 μM, 24 h) and then **Probe 7** (2 μM, 30 min); (**f**) zebrafish cultured by pravastatin (50 μM, 24 h) and further incubated with BSO (200 μM, 12 h) and then **Probe 7** (2 μM, 30 min). Scale bar: 100 μm. Reproduced with permission from ref. [49]. Copyright 2021 American Chemical Society.

In 2021, Chen et al. [49] designed and synthesized a fluorescent probe, **Probe 7**, by combining the OPD part of the BODIPY core at the 5-position as the NO capture indicator and chlorine at the 3-position as the GSH response site. The probe is highly selective and sensitive and detects NO and GSH sequentially, but it can only detect the increase in GSH caused by the presence of NO. As shown in Figure 10A, the probe first reacts with NO, showing yellow fluorescence enhancement at 565 nm, and further activates the reaction site with GSH, showing redshift emission at 595 nm. CCK-8 assay showed that the probe had low toxicity to cells. In addition, the probe visualized NO-induced GSH up-regulation in pravastatin (or VC) treated HUVECs and zebrafish (Figure 10B) for the first time, demonstrating the link between NO and GSH during treatment.

**Figure 11:**
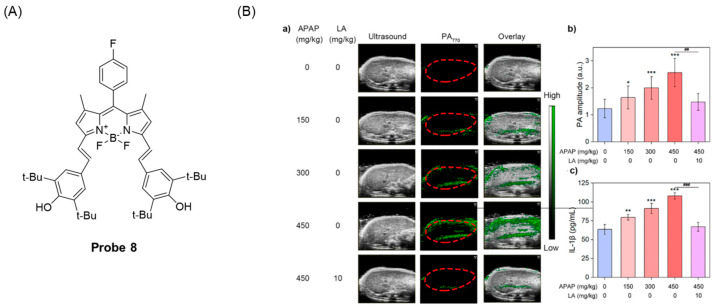
(**A**) Structure of **Probe 8**. (**B**) In vivo PA imaging of ClO^−^/GSH using **Probe 8**. (**a**) Representative PA images of a control mouse liver, different concentrations of the APAP-treated mouse liver and LA-treated ALI mouse liver. (**b**) Changes in the PA770 amplitude of the mice after the intravenous injection of **Probe 8** (n = 3). (**c**) Changes in the IL-1β concentration in serum of mice with different treatments (n = 6). Data are means ± SD. Compared with a control group, * *p* < 0.05, ** *p* < 0.01, and *** *p* < 0.001; compared with a 450 mg/kg APAP-induced group, ^##^ *p* < 0.01 and ^###^ *p* < 0.001. Reproduced with permission from ref. [50]. Copyright 2022 American Chemical Society.

In 2022, Chan et al. [50] reported a case of a reversible near-infrared photoacoustic (PA) probe, **Probe 8**, (Figure 11A) based on the BODIPY fluorophore. The probe is based on the oxidation and reduction process of the 2,6-di-tert-butylphenol group to achieve PA imaging of ClO^−^/GSH. During the selective oxidation of ClO^−^ and reduction recovery of GSH, the absorption of the probe between 648 and 795 nm changes significantly. The probe has high sensitivity and specificity to the ClO^−^/GSH oxygenated reductive loop, and the MTT test and H&E staining test proved that the probe had low toxicity and good biocompatibility. The detection limit of GSH by the probe was 7.2 μM. It was proven that the probe has been successfully applied to the visual measurement of ClO^−^/GSH in the inflammatory microenvironment and ALI model (Figure 11B).

**Figure 12:**
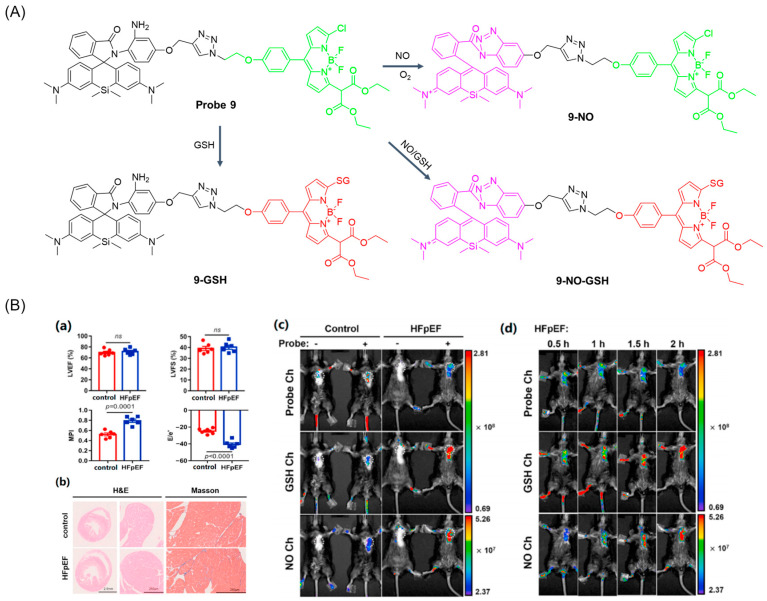
(**A**) Structure of **Probe 9** and its reaction with GSH and NO. (**B**) Validation of a HFpEF mouse model and in vivo fluorescence imaging of cardiac NO and GSH in HFpEF mice. (**a**) Cardiac diastolic dysfunction in HFpEF mice revealed by echocardiography. LVEF, left ventricle ejection fraction; LVFS, left ventricular fractional shortening; MPI, myocardial performance index; E/e′, ratio between mitral E wave and e′ wave (E, peak Doppler blood inflow velocity across mitral valve during early diastole; e′, peak tissue Doppler of myocardial relaxation velocity at mitral valve annulus during early diastole) (n = 5 mice per group, ns, not significant). (**b**) Representative images of hematoxylin & eosin (H&E) and Masson’s Trichrome (MT) staining in transversal sections of heart after 15 weeks of diet. (**c**) Fluorescence images of normal or HFpEF model mice which were preinjected without or with **Probe 9** (40 μM) for 2 h. (**d**) Fluorescence images of HFpEF model mouse after tail intravenous injection of **Probe 9** (40 μM) at different time points. All of fluorescence images were acquired by an IVIS spectrum imaging system through three different sets of channels (λ_ex_ = 500 nm, λ_em_ = 520 nm; λ_ex_ = 535 nm, λ_em_ = 560 nm; λ_ex_ = 640 nm, λ_em_ = 680 nm). Reproduced with permission from ref. [51]. Copyright 2022 Elsevier.

In 2022, Chen et al. [51] developed a case of a fluorescent probe, **Probe 9**, containing both rhodamine and BODIPY fluorophores for simultaneous and independent detection of NO and GSH. As shown in Figure 12A, the probe itself had a green fluorescence response at 525 nm, and after adding GSH, the chlorine on the fluorophores BODIPY was replaced by a “SNAR-rearrangement” mechanism, opening the redshift fluorescence emission channel. The fluorescence intensity of the fluorescence emission at 558 nm had a good linear relationship with the concentration of GSH in the range of 0 to 50 μM, and the detection limit was 2.3 × 10^−7^ M. The probe showed high selectivity and sensitivity to NO and GSH under physiological pH conditions. Cell imaging showed that the probe had low cytotoxicity and good biocompatibility. Moreover, imaging of GSH and NO with this probe clearly showed cardiac oxidative and nitrifying stress in the hearts of heart failure with preserved ejection fraction (HFpEF) mice (Figure 12B), promising to distinguish between normal and HFpEF mice. The probe provides a molecular imaging tool that may help guide and improve the treatment of HFpEF.

In this section, **Probes 1**–**9** are the focus of discussion for BODIPY fluorophore, and Table 1 summarizes the selectivity, sensitivity, and applications of these probes. **Probes 1**, **2**, **7**, **8**, and **9** use dual-channel or three-channel detection of GSH and other substances. **Probes 3** and **4** are specific for GSH recognition and have advantages in sensitivity. **Probes 5** and **6** recognize GSH specifically and are reversible, restoring the original fluorescence of the probe in the presence of H_2_O_2_ or NEM.

### 2.2. Fluorescent Probe Based on 1,8-Naphthalimide

**Figure 13:**
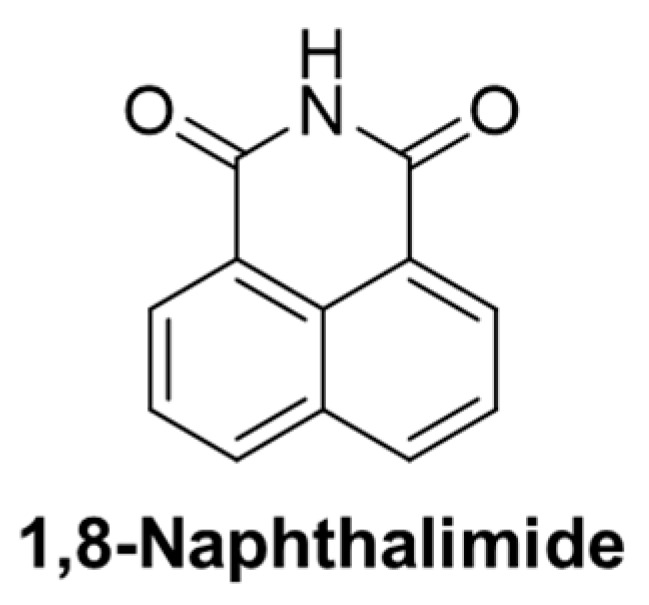
Structure of fluorophore 1,8-Naphthalimide.

The 1,8-Naphthalimide fluorophore (Figure 13) is an ideal scaffold with adjustable photophysical properties, good photostability, and a large Stokes shift [52]. Sensory properties of 1,8-Naphthalimide-based materials include photoinduced electron transfer (PET), intramolecular charge transfer (ICT), fluorescence resonance energy transfer (FRET), and aggregation-induced emission quenching or enhancement (AIEQ/AIEE). 1, 8-Naphthalimide fluorophore structure is easily modified and has synthetic versatility, which is widely used in the construction of probes [53].

**Figure 14:**
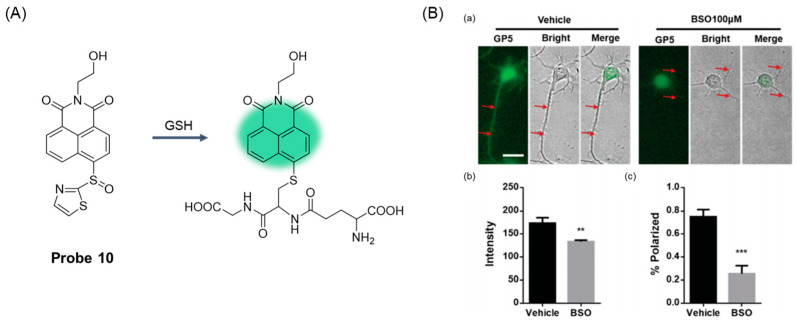
(**A**) Structure of **Probe 10** and its reaction with GSH. (**B**) Inhibiting GSH synthesis suppressed neuronal polarization in cultured primary cortex neurons. (**a**) Isolated primary cortex neurons from E16. The cortexes of 5-day-old mice were cultured in vitro and treated with BSO (100 mM) for 40 h followed by staining with **Probe 10** for 30 min. The axons are highlighted by arrows. (**b**) Quantified fluorescence intensities of cells as represented in panel (**a**). (**c**) Quantified percent of primary cortex neurons with the successful extension of a typical axon on neuronal polarization. Results are presented as mean ± S.D. Statistical significance was determined at *p* < 0.01 (**); *p* < 0.001 (***). Reproduced with permission from ref. [54]. Copyright 2020 Creative Commons.

To improve the sensitivity of the probe to GSH detection, in 2020, Zong et al. [54] developed a GSH super-sensitive probe, **Probe 10**, (Figure 14A) using sulfonyl as a fluorophore, which can detect the subtle GSH fluctuations of developing neurons very sensitively. The probe had excellent selectivity for GSH in living cells. The fluorescence intensity was linearly correlated with GSH concentration in the range of 5–70 μM, and the detection limit was calculated to be 0.11 μM. MTT assay showed that the probe had low cytotoxicity. More importantly, inhibition of GSH was found to delay neuronal polarization by using this probe, revealing for the first time the indispensable role of GSH in neuronal polarization (Figure 14B).

**Figure 15:**
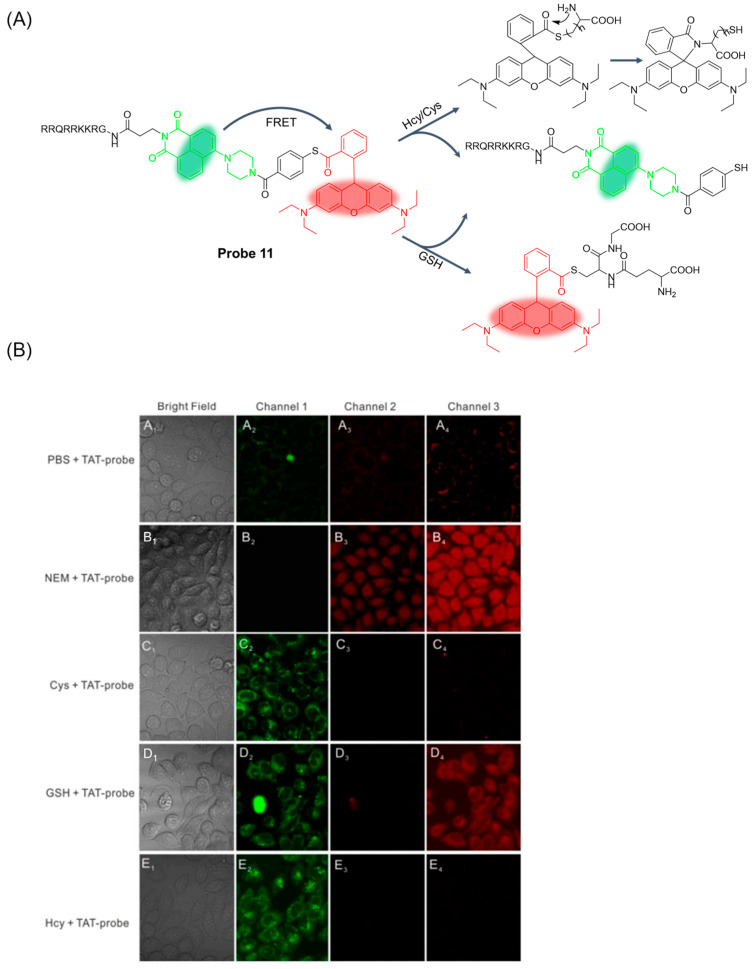
(**A**) Structure of **Probe 11** and its reaction with GSH, Hcy, and Cys. (**B**) Two-photon confocal microscopy fluorescence images of **TAT-probe (Probe 11)** in HeLa cells. Cells treated with the **TAT-probe** (5 μM) (**A**) PBS; (**B**) NEM (1 mM); (**C**) Cys (100 μM); (**D**) GSH (100 μM); (**E**) Hcy (100 μM). (**1**) Bright-field images; (**2**) channel 1, λ_ex_ = 820 nm and λ_em_ = 520 ± 10 nm; (**3**) channel 2, λ_ex_ = 820 nm and λ_em_ = 585 ± 10 nm; (**4**) channel 3, λ_ex_ = 545 nm and λ_em_ = 585 ± 10 nm. Reproduced with permission from ref. [55]. Copyright 2020 Elsevier.

In 2020, Su et al. [55] reported a membrane-penetrating peptide-modified proportional two-photon fluorescence probe, **Probe 11**, which was constructed by the cell penetrating peptide (TAT), 1, 8-naphthalimide, and rhodamine B. As shown in Figure 15A, the probe simultaneously distinguished and sequentially detected Cys, Hcy, and GSH through two different excitation and emission channels. When excited at 404 nm, the probe showed a change in the fluorescence intensity ratio (I_520nm_/I_585nm_) with the addition of thiols. When excited at 545 nm, the probe showed enhanced red fluorescence (λ_em_ = 585 nm) for GSH and fluorescence quenching for Hcy or Cys. This specific fluorescence response indicates that the probe can efficiently detect thiols and distinguish between GSH and Cys/Hcy in mitochondria. The detection limit of the probe for GSH was 5.15 μM. In addition, the time-dependent emission spectra showed that the reaction process was completed within 20 min, and the probe had a fast reaction speed. Moreover, the probe was shown to have low toxicity by the MTT test and was experimentally studied in HeLa cells (Figure 15B).

**Figure 16:**
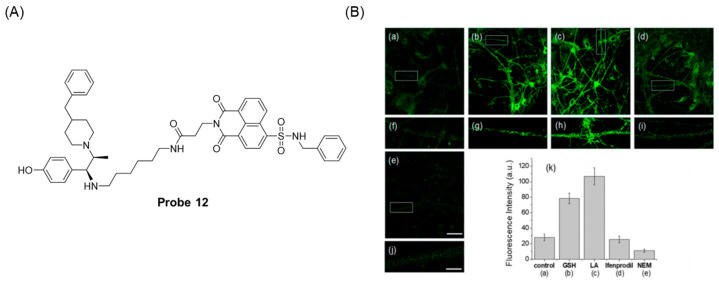
(**A**) Structure of **Probe 12**. (**B**) TPM images of 10 μM **Probe 12** stained primary cortical neuronal cells for 30 min. TPM images of pretreatment without (**a**) and with 500 μM GSH for 1 h (**b**), 500 μM lipoic acid for 24 h (**c**), 10 μM ifenprodil for 30 min (**d**), and 100 μM NEM for 20 min (**e**). (**f**–**j**) Enlarged images of the white box (dendritic spine) in (**a**–**e**). (**k**) Relative TPEF intensity bar graph. The TPEF intensities were collected at 450−600 nm under TP excitation: 750 nm (fs pulses). Each cell represented images from five independent replicate experiments. Scale bars: (**a**–**f**) 60 μm and (**g**–**j**) 15 μm, respectively. Reproduced with permission from ref. [56]. Copyright 2021 American Chemical Society.

In 2021, Kwon et al. [56] reported a case of a GSH-specific fluorescent probe, **Probe 12**, capable of targeting the N-methyl-D-aspartate (NMDA) receptor. As illustrated in Figure 16A, the probe consisted of 1,8-Naphthalimide as a two-photon fluorophore, sulfonamide as a GSH reactive group, and an interferon component as a guide group for the NMDA receptor. After the addition of GSH and Cys/Hcy, the probe showed high selectivity to GSH and showed significant fluorescence enhancement at 495 nm. The detection limit of GSH by the probe was 9.3068 × 10^−3^ M. In addition, the probe has been successfully used for GSH monitoring in neuronal cells (Figure 16B) and rat hippocampal tissue slices under 750 nm excitation of two-photon fluorescence microscopy.

**Figure 17:**
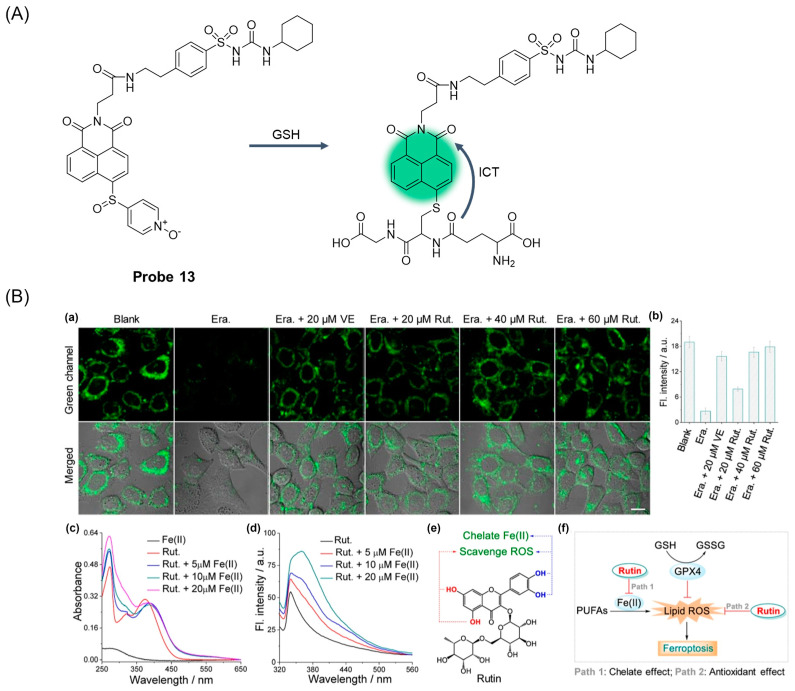
(**A**) Structure of **Probe 13** and its reaction with GSH. (**B**) Fluorescence images of the HeLa cells untreated (blank group), treated with 10 μM erastin for 4 h (Era. group), treated with 10 μM erastin and 20 μM VE for 4 h (Era. + 20 μM VE group) or treated with 10 μM erastin and 20–60 μM rutin for 4 h (Era. + Rut. group) and then stained with 5 μM **Probe 13** for 20 min. (**b**) Quantified fluorescence intensity in green channels in (**a**) using ImageJ software (ImageJ). The error bars represent standard deviation (±SD). Green channel: λ_ex_ = 405 nm and λ_em_ = 500–550 nm. Scale bar = 10 μm. (**c**) Absorption spectra of 5 μM rutin treated with Fe(II) ions. (**d**) Fluorescence spectra of 5 μM rutin treated with Fe(II) ions. λ_ex_ = 300 nm. (**e**) Roles of the phenolic hydroxyl groups at rutin during ferroptosis. (**f**) Proposed inhibition mechanism of rutin to ferroptosis. Reproduced with permission from ref. [57]. Copyright 2023 American Chemical Society.

In 2023, Chen et al. [57] designed and synthesized an endoplasmic reticulum targeting fluorescent probe, **Probe 13**, (Figure 17A) using 1,8-Naphthalimide as fluorophore, and the recognition mechanism is due to the ICT effect. After adding GSH, the probe showed obvious fluorescence enhancement at 523 nm, and the fluorescence intensity showed a good linear relationship with the concentration of GSH in the range of 0–400 μM; the detection limit was 0.12 μM. The probe showed high sensitivity and selectivity to GSH. The probe can be used for visual monitoring of GSH in living cells under a physiological pH environment (pH = 7.0–9.0) and has low cytotoxicity. The probe has excellent endoplasmic reticulum targeting ability and can detect the level of GSH in the endoplasmic reticulum during erastin-induced ferroptosis (Figure 17B).

**Figure 18:**
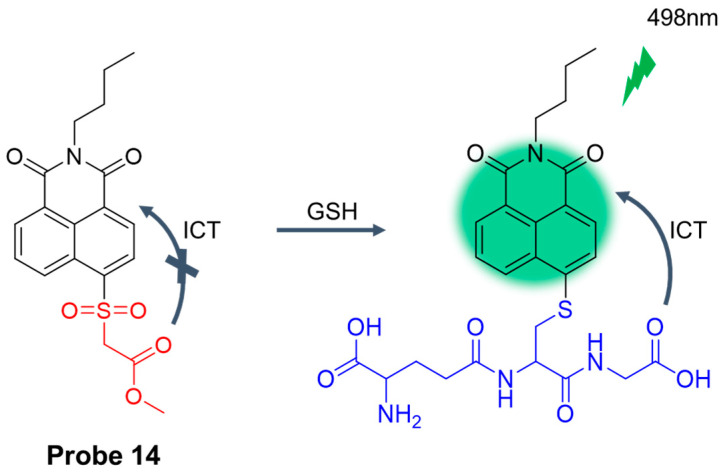
Structure of **Probe 14** and its reaction with GSH. Reproduced with permission from ref. [58]. Copyright 2023 American Chemical Society.

In 2023, Zhang et al. [58] designed and synthesized a nucleophilic aromatic substituted fluorescent probe, **Probe 14**, based on naphthyl amine skeleton. The accuracy of the probe in quantifying GSH in cells and tissues was comparable to that of HPLC. As shown in Figure 18, after adding GSH, the probe showed obvious fluorescence enhancement at 498 nm. The probe had a clear response to GSH within the physiological pH. The probe has the advantages of high reaction activity, selectivity, stability, and low cell toxicity. The probe can accurately quantify the GSH content in the liver of mice with X-rays, reveal a significant reduction in GSH in the liver, and indicate that the fluctuations in GSH content can be seen as indicators of oxidative stress induced by radiation. In addition, the GSH content was quantitatively measured by the probe on the GSH content of the mice in the Parkinson’s disease (PD) model, and the results showed that the content of GSH could reflect the ability of the body to clear free radicals.

In this section, **Probes 10**–**14** are the focus of discussion for 1,8-Naphthalimide fluorophore, and Table 2 summarizes the selectivity, sensitivity, and applications of these probes. **Probes 10**, **13**, and **14** are highly selective for GSH recognition, and **Probe 11** is a dual-channel discriminator for GSH and Cys/Hcy.

### 2.3. Fluorescent Probe Based on Coumarin

**Figure 19:**
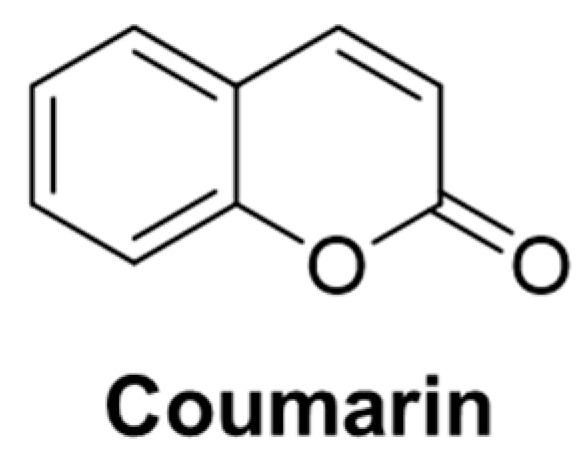
Structure of fluorophore coumarin.

Coumarin (Figure 19), also known as 1,2-benzopyranone and o-xanaphthone, has the advantages of adjustable optical properties, easy synthesis, excellent cell membrane permeability, and favorable biocompatibility [59]. Fluorescent probes using coumarin as fluorophores have become the imaging tool of choice for real-time and non-invasive monitoring of target analytes in biological systems [60].

**Figure 20:**
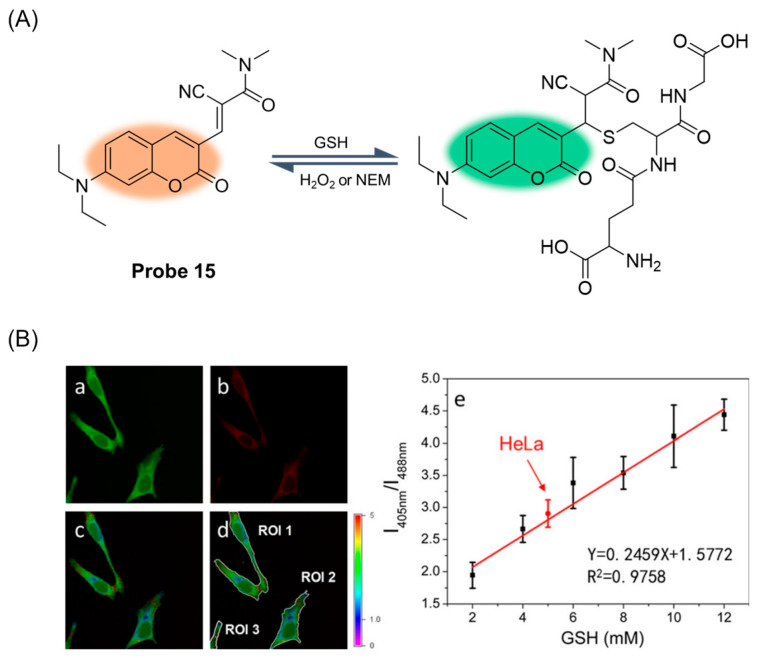
(**A**) Structure of **Probe 15** and its reaction with GSH. (**B**) Measurements of GSH levels in HeLa cells using ratiometric fluorescence imaging. Representative images of HeLa cells treated with **Probe 15** (10 μM) from the green (**a**) and red (**b**) channels. (**c**) The ratiometric image from the green and red channels, which represents the distribution of GSH levels, colored according to the ratio scale shown on the right. (**d**) The regions of interest (ROI) from (**c**). (**e**) Fluorescence intensity ratios as a function of GSH concentration were produced using the same instrument setting as the live cell imaging experiment. The data point in red represents the natural GSH concentration in HeLa cells. Error bars represent standard deviations. Reproduced with permission from ref. [61]. Copyright 2017 John Wiley and Sons.

In 2017, Liu et al. [61] developed a ratio fluorescence probe, **Probe 15**, (Figure 20A) for quantitative monitoring of cellular GSH. Referring to previous studies, they accelerated the Michael addition reaction by introducing a strong electron-withdrawing substituent (CN) into the α-carbon of the probe’s unsaturated carbonyl group. After the addition of GSH, the fluorescence peak at 560 nm decreased, and the new peak at 488 nm gradually increased, showing a proportional fluorescence response. The probe had a suitable dissociation constant (K_d_ = 2.59 mM) and fast response time (t_1/2_ = 5.82 s). It has been proven that the probe has very low cytotoxicity and high selectivity to GSH. In addition, the probe was applied to determine the concentration of GSH in living HeLa cells (5.40 ± 0.87 mM) (Figure 20B) and was able to monitor the changes in GSH/H_2_O_2_ redox pairs in the cells in real time.

**Figure 21:**
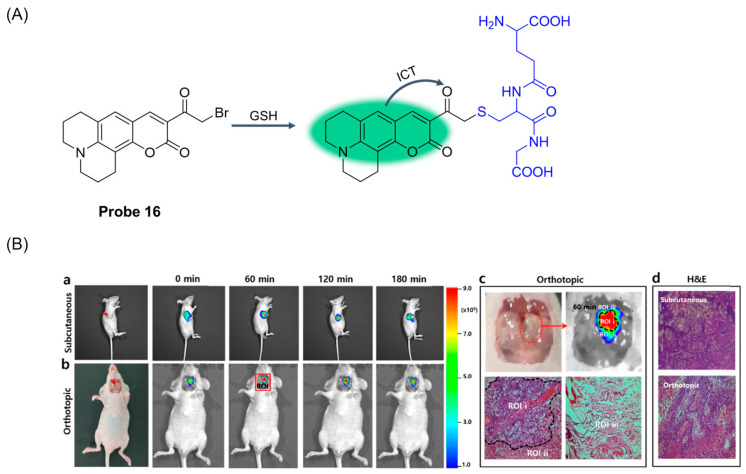
(**A**) Structure of **Probe 16** and its reaction with GSH. (**B**) Time-dependent fluorescence images in subcutaneous and orthotopic laryngeal carcinoma mice models (λ_ex_ = 480 nm, λ_em_ = 500–550 nm). (**a**) Fluorescence images of GSH with **Probe 16** (intertumoral injection, 50 μM, 50 μL in DMSO/saline = 1:99, *v*/*v*) in subcutaneous laryngeal cancer mice model. (**b**) Fluorescence images of GSH with **Probe 16** (local spray, 50 μM, 50 μL in DMSO/saline = 1:99, *v*/*v*) in orthotopic laryngeal cancer mice model. The red arrows indicated the location of subcutaneous and orthotopic tumor, respectively. (**c**) Region of interest (ROI): the imaging and H&E staining of orthotopic tumor with **Probe 16** for 60 min. (**d**) H&E staining of subcutaneous and orthotopic laryngeal cancer tissue, respectively. Reproduced with permission from ref. [62]. Copyright 2020 American Chemical Society.

To further explore whether GSH can be used as a bioindicator to identify tumor lesions at the cellular level, Zou et al. [62] developed a two-photon fluorescence probe, **Probe 16**, based on coumarin derivation 16 in 2019. As shown in Figure 21A, after adding GSH, the probe showed an obvious fluorescence emission at 520 nm, and an ICT effect occurred in the recognition process. After recognition, the fluorescence intensity increased with the increase in GSH concentration, and the detection limit was 90 μM. The probe maintained a relatively stable pH range and had the advantages of low cytotoxicity and high biocompatibility. The probe can detect GSH sensitively and selectively in complex biological systems with a fluorescence quantum yield of 0.375. In addition, the application of the probe in a tumor-bearing mouse model demonstrated its in vivo bioimaging and tumor recognition capabilities (Figure 21B). The probe has been successfully used to determine the surgical margin between tumor and non-tumor tissue in fresh laryngeal cancer specimens.

**Figure 22:**
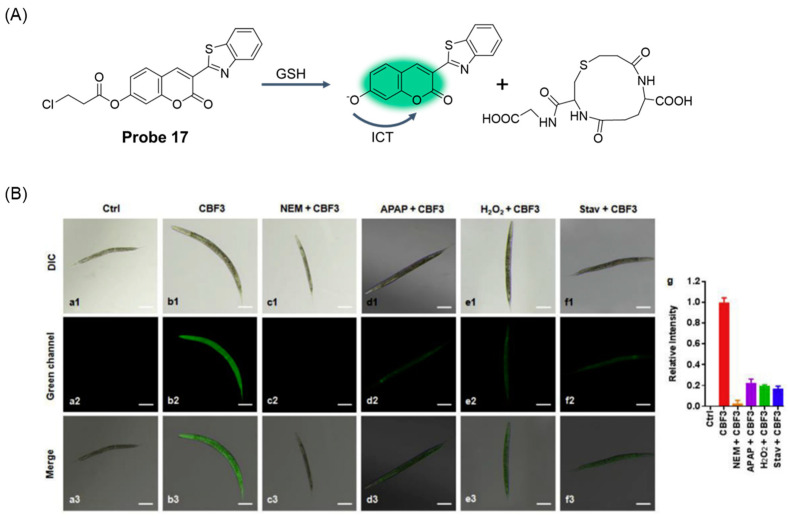
(**A**) Structure of **Probe 17** and its reaction with GSH. (**B**) Fluorescence imaging of GSH using **CBF3 (Probe 17)** in *C. elegans*. (**a**) Control. (**b**) *C. elegans* treated with **CBF3** (10 μM) for 1 h. (**c**) *C. elegans* pretreated with NEM (1 mM) for 30 min and then treated with **CBF3** (10 μM) for 1 h. *C. elegans* pretreated with (**d**) APAP (25 mM) or (**e**) H_2_O_2_ (50 μM) for 30 min and then treated with **CBF3** (10 μM) for 1 h. (**f**) *C. elegans* incubated without food for 12 h and then treated with **CBF3** (10 μM) for 1 h. (**g**) The relative intensity of **CBF3** in different groups. (**1**) Bright field; (**2**) green channel (460–550 nm); (**3**) merged images of (**1**) and (**2**). Scale bar = 20 μm. Stav = Starvation. Reproduced with permission from ref. [63]. Copyright 2020 Elsevier.

In 2020, Li et al. [63] developed a coumarin-based fluorescent probe, **Probe 17**, (Figure 22). The reaction mechanism was confirmed by LC-MS and NMR analysis and density functional theory calculations such as nucleophilic substitution/cyclic and ICT. The probe showed high selectivity, high sensitivity, and good stability for GSH detection. With the increase in GSH concentration, the fluorescence of the probe at 495 nm was gradually enhanced, showing a good linear relationship. The fluorescence quantum yield of the probe for GSH was 0.85, and the detection limit was 9.2 nM. In addition, the probe can quantitatively detect GSH not only in serum samples but also in living cells and *C. elegans* under various oxidative stress conditions.

**Figure 23:**
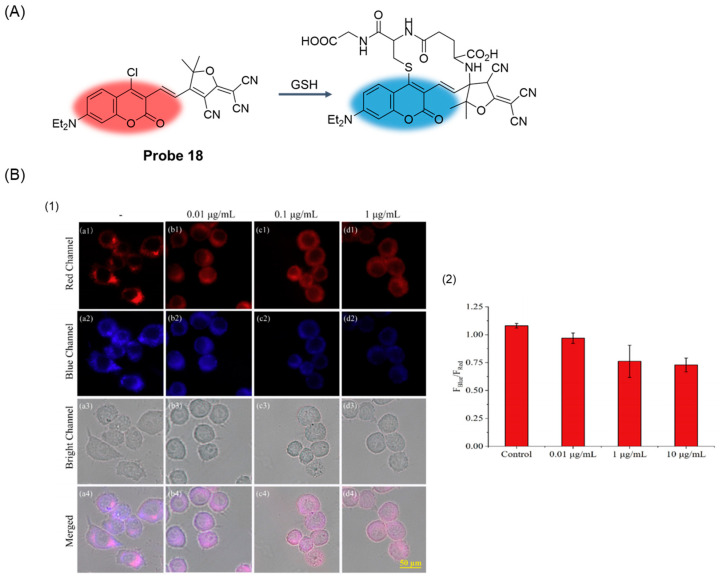
(**A**) Structure of **Probe 18** and its reaction with GSH. (**B**) (**1**) Fluorescence images of **Probe 18** with LPS in Raw 264.7 cells. (**a**–**d**) Cells were treated with 0.01, 0.1, and 1 μg/mL LPS for 24 h then incubated with 5 μM **Probe 18** for 30 min. The fluorescence images were captured under the red channel of 650–750 nm and blue channel of 430–490 nm with excitation of 575 nm and 415 nm, respectively. 1: Red Channel; 2: Blue Channel; 3: Bright Channel; 4: Merged. Scale bar: 50 μm. (**2**) Fluorescence intensity ratios (F_Blue_/F_Red_) in panels a–d. Reproduced with permission from ref. [64]. Copyright 2020 Elsevier.

In 2020, Niu et al. [64] designed and synthesized a novel fluorescent probe, **Probe 18**, (Figure 23A) characterized by chlorinated coumarin-TCF through the condensation reaction of chlorinated coumarin-aldehyde and TCF. Under physiological pH conditions, the probe can detect GSH with high selectivity and sensitivity. After the addition of GSH, the fluorescence of the probe at 471 nm is enhanced, and the relationship is linear with the concentration of GSH in the range of 0–40 μM, and the detection limit is calculated to be 5.5 nM. CCK-8 assay proved that the probe had low toxicity to cells. In addition, the probe can be used to observe endogenous and exogenous GSH in different kinds of cells through fluorescence imaging. It has also been successfully used to monitor cellular GSH dynamics caused by H_2_O_2_ and LPS-induced redox imbalances (Figure 23B).

**Figure 24:**
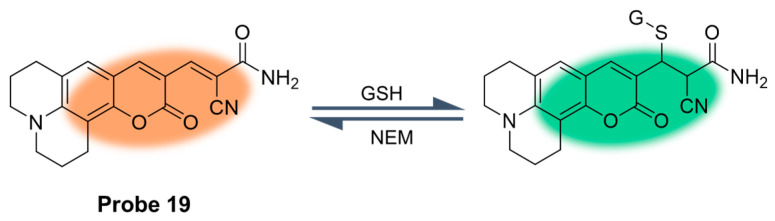
Structure of **Probe 19** and its reaction with GSH. Reproduced with permission from ref. [65]. Copyright 2020 American Chemical Society.

In 2020, Tian et al. [65] developed a rate-fluorescence probe, **Probe 19**, based on a coumarin Michael addition reaction. The reaction rate of the probe with GSH was very fast, as shown in Figure 24, and a reversible reaction occured after NEM treatment. The addition of GSH to the probe produced a fluorescence emission peak at 505 nm, while the original emission peak at 587 nm was weakened. The ratio of fluorescence intensity F_505_/F_587_ increased with the concentration of GSH from 0.1 to 10 mM. The probe showed high selectivity to GSH and low toxicity by an MTT test. In addition, the probe was successfully applied to real-time monitoring of GSH levels in living cells. Furthermore, an image processing program was developed and validated to quantify fluorescence intensity in cells.

**Figure 25:**
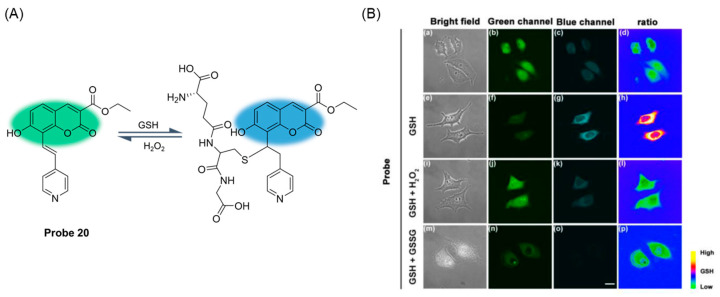
(**A**) Structure of **Probe 20** and its reaction with GSH. (**B**) Confocal fluorescence images of HeLa cells, which were incubated with probe **Probe 20** (5 μM) for 1 h. (**a**–**c**): only with **Probe 20** (5 μM); (**e**–**g**): **Probe 20** (5 μM) pretreated cells incubated with GSH (6 mM) for 10 min; (**i**–**k**): **Probe 20** (5 μM) pretreated cells treated with GSH (6 mM) for 10 min then with H_2_O_2_ (6 mM) for 10 min; (**m**–**o**): **Probe 20** (5 μM) pretreated cells treated with GSH (6 mM) for 10 min then with GSSG (6 mM) for 10 min. The first column is the bright-field image; the second column is the green channel image (collected at 510–540 nm, λ_ex_ = 410 nm); the third column is the blue channel image (collected at 425–475 nm, λ_ex_ = 350 nm); (**d**,**h**,**l**,**p**) the last column is the pseudocolor ratio (blue/green). Scale bar: 20 μm. Reproduced with permission from ref. [66]. Copyright 2020 American Chemical Society.

In 2020, Khatun et al. [66] developed a ratio fluorescence probe, **Probe 20**, based on coumarin fluorophore for monitoring GSH levels in the nucleus. In the presence of GSH, the probe showed a change in the ratio of the ultraviolet absorption spectrum to the emission spectrum, which shifted from 410 nm to 350 nm blue, and the fluorescence emission spectrum shifted from 510 nm to 460 nm blue. As shown in Figure 25A, in the presence of reactive oxygen analogs GSSG and H_2_O_2_, Michael’s addition of GSH to the probe was reversible. The probe had a dissociation constant (K_d_) of 2.47 mM under physiological pH conditions. The probe showed the advantages of high selectivity and low toxicity to GSH. In addition, the probe was able to specifically target GSH in the nucleolus of living cells (Figure 25B), suggesting that the probe could be a useful tool for tracking GSH during the cell cycle.

**Figure 26:**
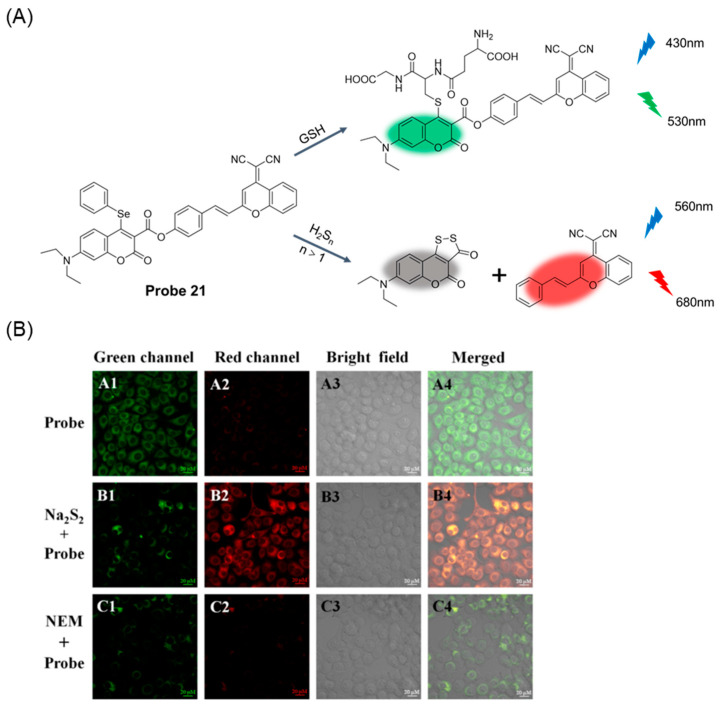
(**A**) Structure of **Probe 21** and its reaction with GSH and H_2_S_n_ (n > 1). (**B**) Fluorescence images of GSH and H_2_S_n_ in living MGC-803 cells. (**A1**–**A4**) Cells only incubated with **Probe 21** (10.0 μM) for 30 min; (**B1**–**B4**) Cells treated with Na_2_S_2_ (220.0 μM) for 15 min and then incubated with **Probe 21** (10.0 μM) for 30 min; (**C1**–**C4**) NEM-pretreated cells incubated with **Probe 21** (10.0 μM) for 30 min. Green channel: λ_ex_ = 405 nm, emissions were collected at 500–550 nm. Red channel: λ_ex_ = 543 nm, emissions were collected at 650–700 nm. Reproduced with permission from ref. [67]. Copyright 2021 Elsevier.

In 2021, Niu et al. [67] developed a coumarin-based long-wavelength emitting fluorescent probe, **Probe 21**. As shown in Figure 26A, the probe can distinguish between GSH and hydrogen polysulfides (H_2_S_n_, n > 1). With the addition of GSH, the probe showed a green fluorescence emission at 530 nm (λ_ex_ = 430 nm). In the presence of H_2_S_n_, the probe exhibited enhanced red fluorescence at 680 nm (λ_ex_ = 560 nm). The probe had good stability, selectivity, and high sensitivity. The probe could quantitatively detect GSH and H_2_S_n_ with detection limits of 0.12 μM and 0.19 μM, respectively. The MTT test proved that the probe had low toxicity to living cells. In addition, the probe can be used to detect endogenous GSH and H_2_S_n_ in living cells (Figure 26B) by two-color fluorescence imaging.

**Figure 27:**
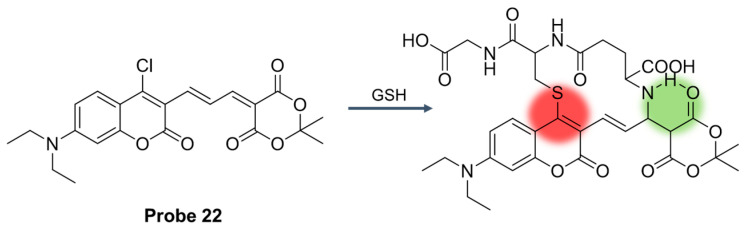
Structure of **Probe 22** and its reaction with GSH. Reproduced with permission from ref. [68]. Copyright 2021 Royal Society of Chemistry.

In 2021, Liu et al. [68] developed a coumarin-based, double-reactive site fluorescent probe, **Probe 22**. The probe reacted with the sulfhydryl or amino group of mercaptan through the S_N_Ar process. As illustrated in Figure 27, the initial fluorescence of the probe was quenched after GSH was added, and a new fluorescence signal appeared at 547 nm. The concentration of GSH was in the range of 0–25 μM, the fluorescence intensity was linear with the concentration, and the detection limit was 0.16 μM. The probe showed high selectivity and specificity for GSH. In the pH 6.0–9.0 range, the probe could label changes in GSH concentration in living cells and tissue sections with a fluorescent response.

**Figure 28:**
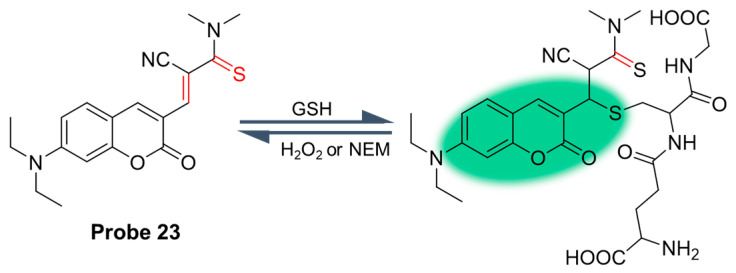
Structure of **Probe 23** and its reaction with GSH. Reproduced with permission from ref. [69]. Copyright 2022 Elsevier.

In 2022, Hou et al. [69] developed a reversible fluorescent probe, **Probe 23**, based on coumarin for real-time quantitative monitoring of GSH in cells. As shown in Figure 28A, after adding GSH, the fluorescence of the probe was enhanced at 486 nm. The fluorescence intensity showed a good linear relationship with GSH concentration in the range of 0–6 mM, and the detection limit was 4.5 μM. With the depletion of GSH and the addition of H_2_O_2_, the probe reformed and showed a reversible fluorescence signal. The probe could detect GSH under physiological conditions, and had good stability, high selectivity, and very low toxicity. In addition, the probe effectively imaged GSH changes in living HeLa cells and determined the GSH concentration in HeLa cells to be 4.25 mM (Figure 28B).

**Figure 29:**
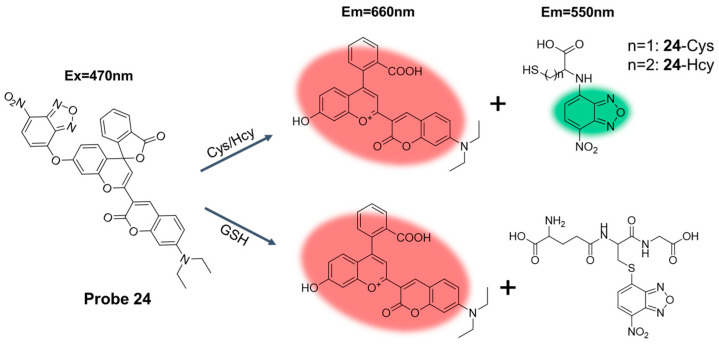
Structure of **Probe 24** and its reaction with GSH, Hcy, and Cys. Reproduced with permission from ref. [70]. Copyright 2023 Elsevier.

In 2022, Liu et al. [70] designed and synthesized a near-infrared fluorescence probe, **Probe 24**, using coumarin as a fluorophore. As illustrated in Figure 29, at 470 nm excitation, the probe was able to distinguish separated GSH/H_2_S and Cys/Hcy from dual emission channels. After the addition of GSH, the probe observed a significant fluorescence enhancement at 660 nm, and the color of the solution changed from yellow to light blue. In addition, the fluorescence intensity had a good linear correlation with the concentration of GSH in the range of 0 to 20 μM, and the detection limit was 0.199 μM. After adding the identifier to the probe, the fluorescence emission peak almost did not change within 7 days, showing high stability. Moreover, the probe had the advantages of high sensitivity, high specificity, and low cytotoxicity. In addition, the probe was successfully applied to distinguish GSH/H_2_S and Cys/Hcy in living cells and zebrafish.

**Figure 30:**
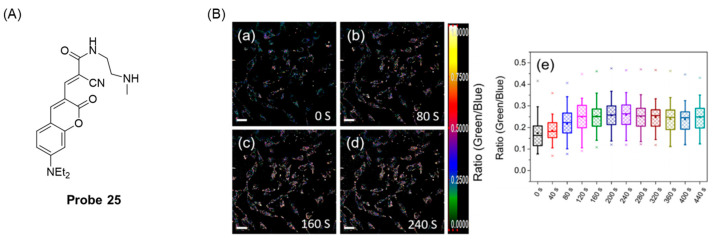
(**A**) Structure of **Probe 25**. (**B**) (**a**–**d**) Representative ratiometric images (green/blue) displaying real-time changes in GSH in HeLa cells treated with NMM (5 mM) (scale bar: 30 μm). (**e**) The fluorescence intensity quantitative analysis of GSH dynamic changes in individual HeLa cells (n = 30). Reproduced with permission from ref. [71]. Copyright 2023 Royal Society of Chemistry.

In 2022, Liu et al. [71] synthesized a ratio fluorescence probe, **Probe 25**, (Figure 30A) for detecting GSH using coumarin as a fluorophore. However, the smaller the K_d(GSH)_, the stronger the affinity between the double bond and the sulfhydryl group. The molecular binding affinity of the probe to GSH was enhanced through a reversible Michael addition reaction. The K_d(GSH)_ of the probe was 18.54 μM, and the affinity for GSH was 113 times that of coumarin itself. With the addition of GSH, the absorption peak at 566 nm decreased rapidly and the absorption peak at 470 nm increased. The fluorescence change was most obvious in the range of 0–0.5 mM GSH concentration, and the detection limit was 148 nM. In addition, the probe had the advantages of high sensitivity, good selectivity, fast response (forward t_1/2_ = 30 s, reverse t_1/2_ = 111 s), and good pH stability (pH = 6.0–10.0). In addition, the probe achieved dynamic real-time sensing of GSH in living HeLa cells (Figure 30B).

**Figure 31:**
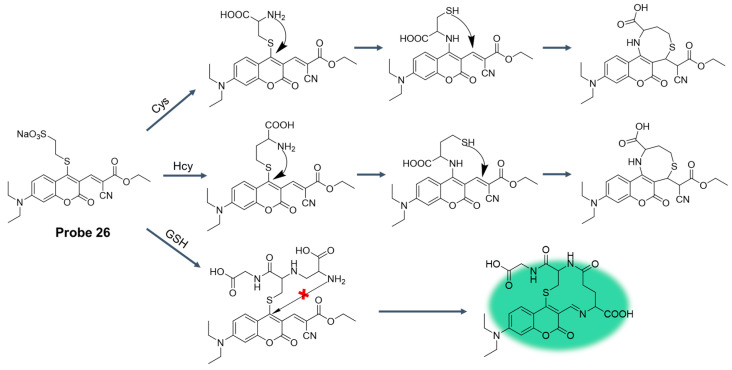
Structure of **Probe 26** and its reaction with GSH, Hcy, and Cys.

In 2023, Du et al. [72] designed and synthesized a fluorescence probe, **Probe 26**, using coumarin as a fluorophore. The introduction of sodium 2-mercaptoethanesulfonate on coumarin as the leaving group increased the water solubility of the probe and provided multiple reaction sites with double bonds. As shown in Figure 31, the probe could detect Cys, Hcy, and GSH in aqueous solution by fluorescence and ultraviolet spectra at different excitation wavelengths. When the excitation wavelength was 490 nm and GSH was added into the probe, the fluorescence color of the solution changed from colorless to light green, and the maximum emission peak appeared at 553 nm. The detection limit of glutathione was 0.92 μM at low concentration. In addition, the probe had good photostability and low cytotoxicity. In addition, the probe has been successfully used in cell experiments to detect endogenous and exogenous biological mercaptans.

In this section, **Probes 15**–**26** are the focus of discussion for coumarin fluorophore, and Table 3 summarizes the selectivity, sensitivity, and applications of these probes. **Probes 16**, **17**, **18**, and **22** can specifically recognize GSH with high sensitivity. **Probes 15**, **19**, **20**, **23**, and **25** are highly selective for GSH recognition and can react reversibly in the presence of H_2_O_2_ or NEM. **Probes 21**, **24**, and **26** are dual-channel or three-channel differentiators that detect GSH and other substances.

### 2.4. Fluorescent Probe Based on Xanthene

**Figure 32:**
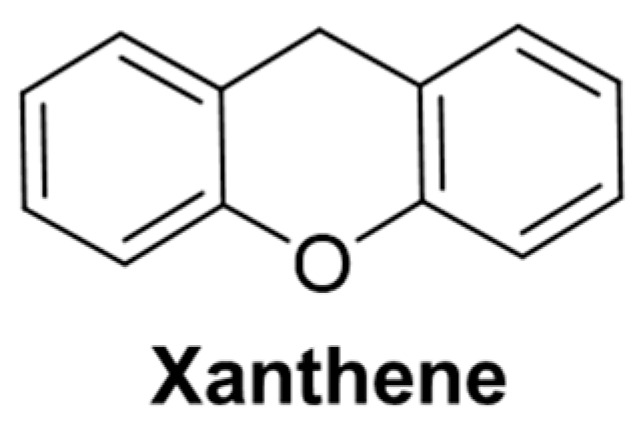
Structure of fluorophore xanthene.

As a photo redox catalyst, xanthene fluorophore (Figure 32) has excellent photophysical properties such as high fluorescence quantum yield, high molar extinction coefficient, high reactivity, good photostability, and chemical stability [73]. Xanthene fluorescent dye is an ideal material for a fluorescence probe skeleton, which is widely used in visible-light-induced organic synthesis [74].

**Figure 33:**
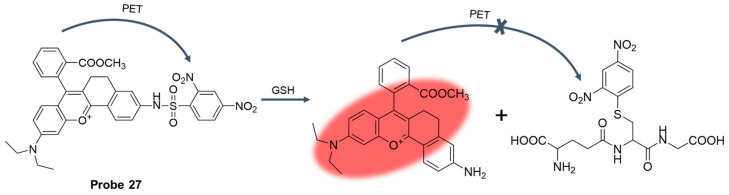
Structure of **Probe 27** and its reaction with GSH. Reproduced with permission from ref. [75]. Copyright 2019 Elsevier.

In 2019, Wan et al. [75] developed a fluorescent probe, **Probe 27**, with xanthene as the fluorophore and DNBS as the recognition site. As shown in Figure 33, the probe blocked the occurrence of PET during the recognition of GSH. With the addition of GSH, the probe had an obvious fluorescence emission at 630 nm. The fluorescence intensity showed a good linear relationship with the concentration of GSH in the range of 1–6 mM, and the detection limit was 13.1 μM. The probe showed good selectivity and anti-interference to GSH and could directly monitor GSH under physiological pH conditions. Moreover, cell activity experiments showed that the probe was biocompatible and had low cytotoxicity. In addition, the probe was successfully applied to fluorescence imaging of living cells.

**Figure 34:**
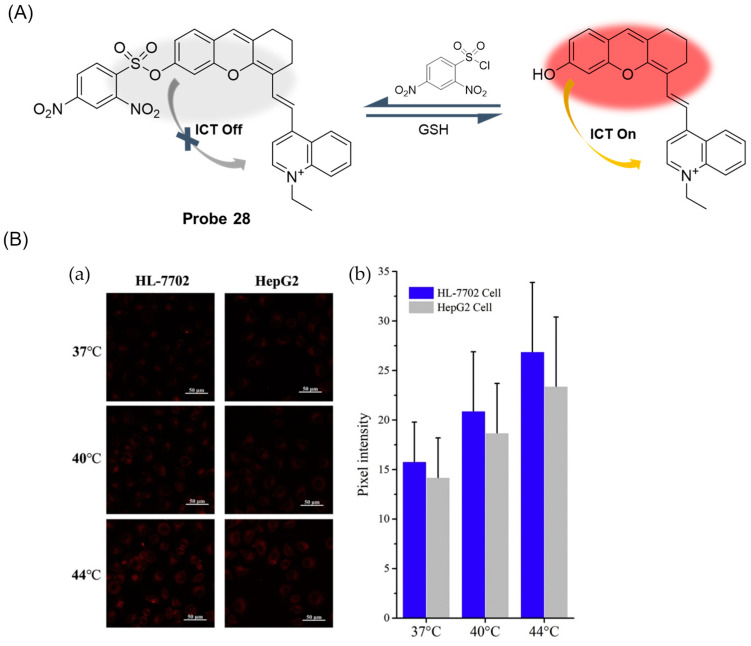
(**A**) Structure of **Probe 28** and its reaction with GSH. (**B**) (**a**) Fluorescence images of GSH in HL-7702 and HepG2 cells at different temperatures (37 °C, 40 °C, and 44 °C). (**b**) Relative fluorescence intensity of fluorescence images. Reproduced with permission from ref. [76]. Copyright 2020 Elsevier.

In 2019, Liang et al. [76] designed and synthesized a water-soluble near-infrared fluorescent probe, **Probe 28**, with DNBS as the recognition unit for sensitive real-time monitoring of changes in intracellular GSH concentration. As shown in Figure 34A, the probe achieved the “on-off” fluorescence response to GSH through the nucleophilic substitution reaction based on the sulfhydryl group. After the addition of GSH, the probe had an obvious fluorescence emission peak at 728 nm, the fluorescence intensity was linear with the concentration of GSH in the range of 0–5 μM, and the detection limit was 69 nM. When pH was 7.0, the fluorescence intensity after probe identification reached the highest, and the fluorescence signal was not affected in the temperature range of 22–50 °C. The probe recognized GSH very quickly, reaching its maximum value within 1 min. Moreover, the probe showed high selectivity for GSH. Finally, cell imaging experiments showed that the probe had low cytotoxicity and was applied to study the fluctuation of GSH concentration in living cells under high-temperature stress (Figure 34B).

**Figure 35:**
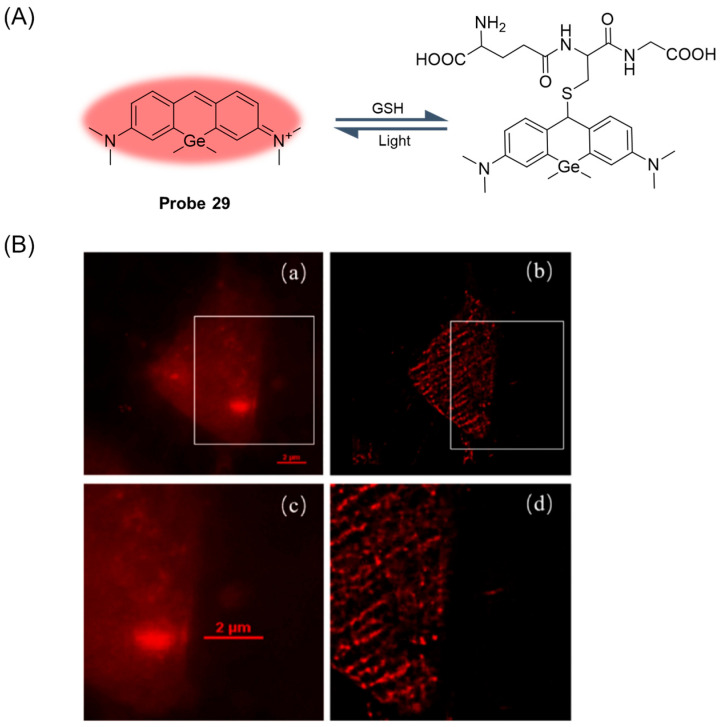
(**A**) Structure of **Probe 29** and its reaction with GSH. (**B**) Stochastic optical reconstruction microscopy (STORM) imaging of mitochondria in HeLa cells. (**a**) Conventional fluorescence image of mitochondria. (**b**) STORM image of the same area. Conventional (**c**) and STORM (**d**) images corresponding to the boxed regions in (**a**,**b**). (λ_act_ = 405 nm, λ_ex_ = 561 nm). Scale bar: 2 μm. Reproduced with permission from ref. [77]. Copyright 2022 Elsevier.

In 2022, Shu et al. [77] designed and synthesized a reversible fluorescent probe, **Probe 29**, by introducing Ge atoms into a conventional O-pyronine (OP) dye. The probe showed a high fluorescence intensity at 644 nm. As illustrated in Figure 35A, after adding GSH, a nucleophilic addition reaction occurred within 1 min and the fluorescence was quenched. Moreover, the adduct dissociated and restored the original fluorescence of the probe under ultraviolet illumination, showing a reversible fluorescence response. There was a good linear relationship between fluorescence intensity and GSH concentration in the range of 0 to 6 μM, and the detection limit was 0.07 μM. The probe could detect intracellular GSH quickly, sensitively, and specifically. Moreover, the probe had the advantages of good spectral characteristics, excellent mitochondrial targeting ability, and good cell permeability, and has been successfully used for the imaging of GSH in mitochondria. In addition, the probe has been successfully applied to super-resolution fluorescence imaging of mitochondria in living cells (Figure 35B).

In this section, **Probes 27**–**29** are the focus of discussion for xanthene fluorophore, and Table 4 summarizes the selectivity, sensitivity, and applications of these probes. **Probes 27**–**29** are highly selective for GSH recognition, and **probes 27** and **28** introduce DNBS in probe construction. **Probes 28** and **29** can react with GSH rapidly, and **probe 29** can react reversibly under light.

### 2.5. Fluorescent Probe Based on Rhodamine

**Figure 36:**
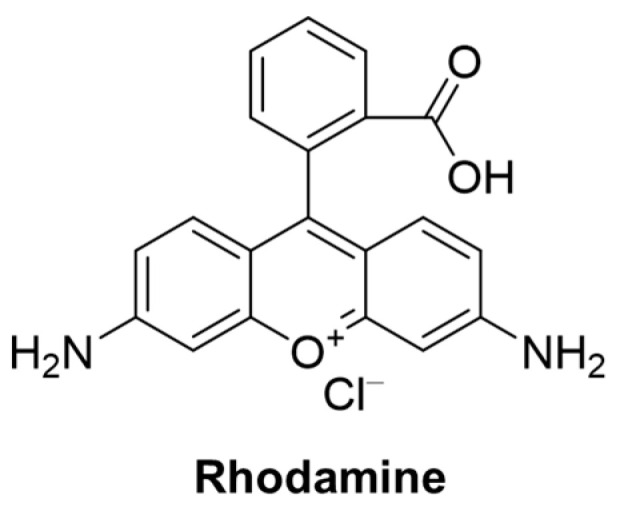
Structure of fluorophore rhodamine.

Rhodamine fluorophore (Figure 36) has been widely used in medical and biological applications due to its unique transition from a helicoid form (non-fluorescent) to an open-loop form (fluorescent), excellent light stability, wide wavelength range, pH insensitivity, high fluorescence quantum yield, and high extinction coefficient [78,79].

**Figure 37:**
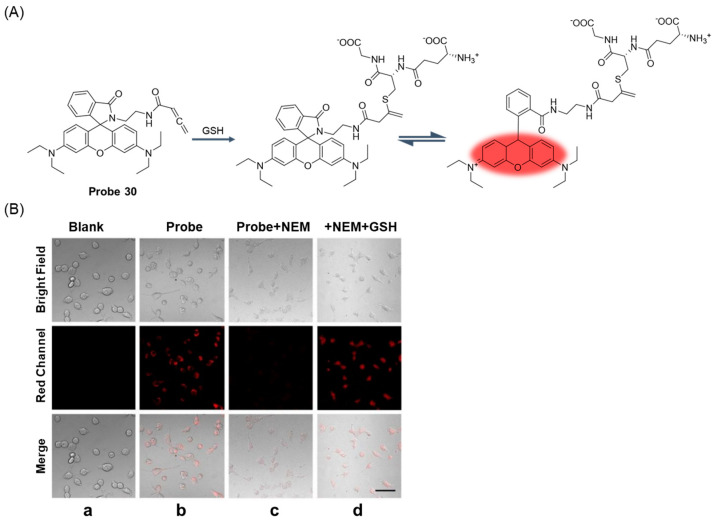
(**A**) Structure of **Probe 30** and its reaction with GSH. (**B**) Confocal images of MCF-7 cells. (**a**) Control MCF-7 cells. (**b**) MCF-7 cells incubated with **Probe 30** only for 5 h. (**c**) MCF-7 cells pre-treated with NEM for 60 min then incubated with **Probe 30** for 5 h. (**d**) MCF-7 cells pre-treated with NEM for 60 min then treated with reduced GSH for 30 min and, finally, incubated with **Probe 30** for 5 h. Scale bar:100 μm. Reproduced with permission from ref. [80]. Copyright 2020 Royal Society of Chemistry.

In 2020, Li et al. [80] developed a fluorescent probe, **Probe 30**, with rhodamine B as a fluorophore for the selective detection of GSH in solution. As illustrated in Figure 37A, the probe turned on fluorescence through a specific reaction between acrylamide and GSH sulfhydryl groups. When GSH was added, the fluorescence of the probe was enhanced at 585 nm. The fluorescence intensity and GSH concentration showed a good linear relationship in the range of 0–20 μM, and the detection limit was 1.0 μM. Moreover, the MTT assay proved that the probe had the advantages of good cell biocompatibility, permeability, and low cytotoxicity. The probe could detect GSH under physiological conditions and showed high selectivity. In addition, the probe could detect changes in GSH concentration in living cells (Figure 37B).

**Figure 38:**
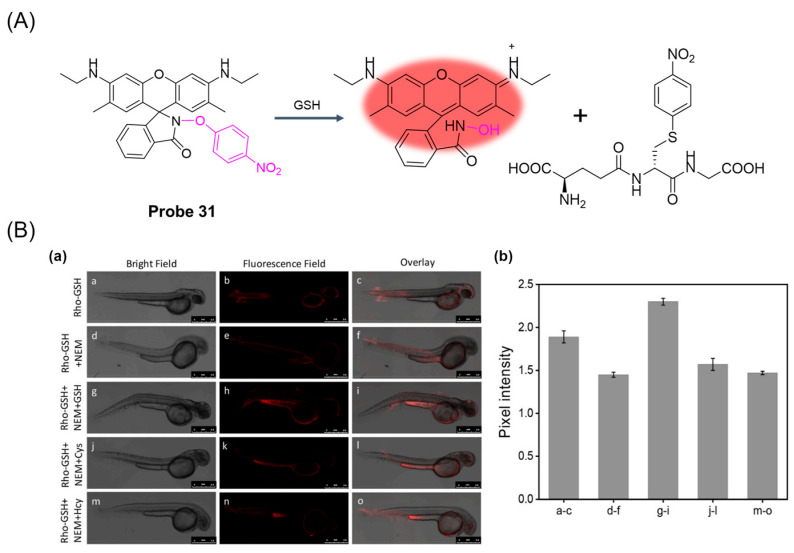
(**A**) Structure of **Probe 31** and its reaction with GSH. (**B**) (**a**) Confocal fluorescence imaging in 3-day-old zebrafish incubated in 10 μM **Rho-GSH (Probe 31)**. Panels (**a**–**c**) show zebrafish incubated with **Rho-GSH** only for 30 min at 37 °C; panels (**d**–**f**) show zebrafish pretreated with 100 μM NEM for 30 min at 37 °C followed by **Rho-GSH** staining; panels (**g**–**i**) show zebrafish pretreated with 100 μM NEM for 30 min at 37 °C then treated with 2 mM GSH followed by **Rho-GSH** staining; panels (**j**–**l**) show zebrafish pretreated with 100 μM NEM for 30 min at 37 °C then treated with 2 mM Cys followed by **Rho-GSH** staining; panels (**m**–**o**) show zebrafish pretreated with 100 μM NEM for 30 min at 37 °C then treated with 2 mM Hcy followed by **Rho-GSH** staining. (**b**) The relative fluorescence intensity graph of zebrafish; λ_ex_/λ_em_ = 514/525–650 nm. Reproduced with permission from ref. [81]. Copyright 2022 Elsevier.

In 2022, Zhou et al. [81] designed and synthesized a rhodamine fluorescent probe, **Probe 31**, with nitrobenzene as the recognition group for monitoring and imaging GSH in vivo and in vitro. As shown in Figure 38A, after the addition of GSH, nucleophilic substitution and helix ring-opening reactions occurred, showing a significant increase in fluorescence at 557 nm. The fluorescence intensity of the probe showed a good linear relationship with GSH concentration in the range of 0–5 mM, and the detection limit was 49.60 μM. The probe showed high selectivity, fast response (3 s), and anti-interference in GSH detection. In addition, the probe was successfully applied to GSH imaging in living cells and zebrafish (Figure 38B), demonstrating the selectivity of GSH imaging relative to Cys and Hcy. The probe was also able to selectively distinguish between cancer cells and normal cells through fluorescence imaging.

**Figure 39:**
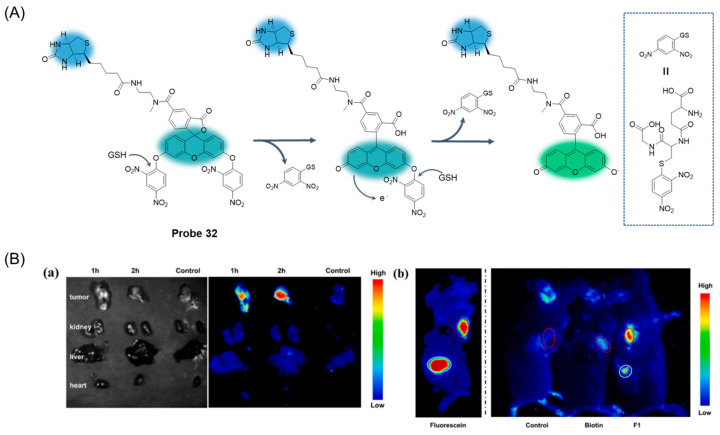
(**A**) Structure of **Probe 32** and its reaction with GSH. (**B**) Fluorescence imaging. (**a**) Representative ex vivo imaging of the tumors and organs of MC38-tumor-bearing C57BL/6J mice 1 or 2 h after intratumoral injection of **F1** (**Probe 32**). (**b**) The difference between fluorescein and **F1** in tumor and normal tissue imaging and verification of the biotin-promoted cellular uptake. Representative in vivo fluorescence images of mice 2 h after intratumoral injection (red circle) and subcutaneous injection (white circle) of **F1** or fluorescein (1 mg/kg). The tumor of biotin verification mice was pre-treated with free biotin (0.5 mg/kg) for 1 h before **F1** injection. All control mice were injected with an equal volume of saline. (n = 3 per group). Reproduced with permission from ref. [82]. Copyright 2022 Elsevier.

In 2022, Lu et al. [82] designed and synthesized a GSH-activated biotin-labeled fluorescent probe, **Probe 32**, for selective colorectal cancer imaging. As shown in Figure 39A, after adding GSH, a nucleophilic reaction occurred between GSH and 2,4-dinitrobenzene, which promoted the activation of the fluorescence effect. When the concentration of GSH was 0 to 300 μM, the fluorescence response was enhanced at 530 nm and showed an obvious linear relationship with a detection limit of 37 μM. Through the CCK8 assay, the probe showed very low toxicity to both cancer cells and normal cells. Imaging of a variety of cells showed that the probe could specifically recognize GSH and showed excellent tumor cell uptake and GSH activation. Moreover, the probe had good tumor tissue imaging ability and good tissue selectivity in MC38 mice (Figure 39B) and could also effectively identify CRC clinical samples. In addition, the study highlights the importance of hydrophobicity in biotin-labeled activable molecules.

In this section, **Probes 30**–**32** are the focus of discussion for rhodamine fluorophore, and Table 5 summarizes the selectivity, sensitivity, and applications of these probes. **Probe 31** can respond to GSH quickly, while **probes 30** and **32** require a two-step reaction to restore fluorescence.

### 2.6. Fluorescent Probe Based on Cyanine

**Figure 40:**
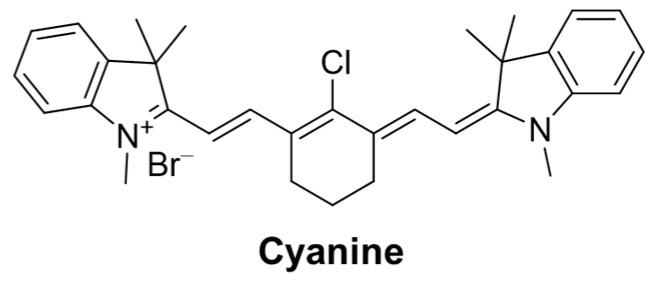
Structure of fluorophore cyanine.

Cyanine dyes (Figure 40) have good optical properties such as a high molar extinction coefficient, high fluorescence quantum yield, long absorption and emission wavelength, good biocompatibility, and low toxicity [83]. Cyanine dyes are widely distributed in the spectrum of approximately 350 nm to 1300 nm, as well as the easily adjustable fluorescence spectrum from the UV-visible to near-infrared range. Therefore, cyanine dyes and their derivatives have outstanding advantages such as fluorescent probes [84].

**Figure 41:**
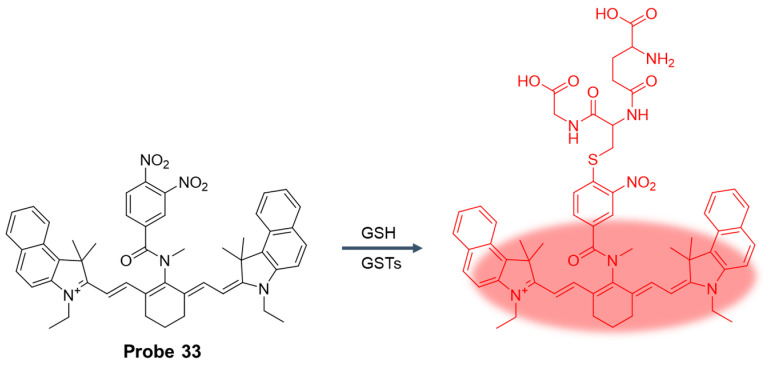
Structure of **Probe 33** and its reaction with GSH. Reproduced with permission from ref. [85]. Copyright 2019 American Chemical Society.

In 2019, He et al. [85] reported a near-infrared fluorescent probe, **Probe 33**, to detect concentration fluctuations of glutathione S-transferase (GST) in cells and mouse models. As shown in Figure 41, GST catalyzes the two-step SNAr addition/elimination mechanism of GSH and the probe, impeding the process of dPET and inducing fluorescence recovery. After the addition of hGSTP1, the fluorescence intensity was significantly enhanced at 810 nm and showed a good linear relationship in the concentration range of 1–1.1 μg/mL, and the detection limit was 10 ng/mL. The fluorescence kinetics curve showed that a high concentration of hGSTP1 could make the fluorescence signal reach a stable level within 15 min, indicating that the probe could respond to hGSTP1 quickly. Under physiological conditions, the probe was incubated with various biological species, and the probe showed excellent selectivity for hGSTP1. Moreover, with the help of this probe, it was confirmed that intracellular GSTs play an important role in the severity of pulmonary fibrosis. Pulmonary fibrosis also leads to a state of intracellular oxidative stress, which also induces an increase in GST levels. In addition, the synergistic treatment of the GST inhibitors TLK117 and pirfenidone (PFD) had a better therapeutic effect in a mouse model of pulmonary fibrosis than the two drugs alone.

**Figure 42:**
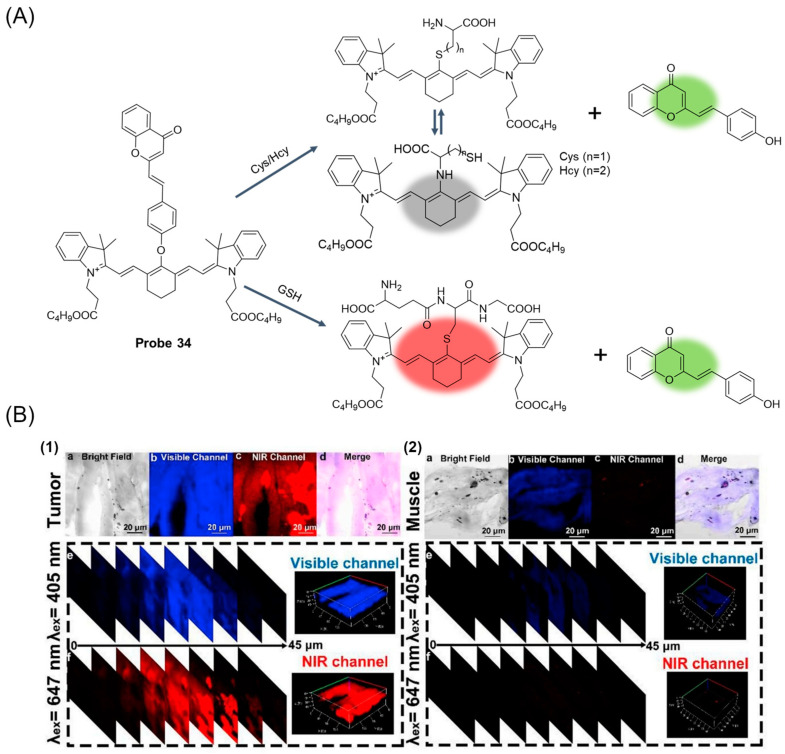
(**A**) Structure of **Probe 34** and its reaction with GSH, Hcy, and Cys. (**B**) (**1**) One-photon images of tumor tissue treated with **Probe 34** (20 μM) for 30 min. (**a**) Bright-field channel of the image. (**b**) The visible channel of the image. (**c**) NIR channel of the image. (**d**) The merged image of (**a**–**c**). (**e**–**f**) The one-photon confocal z-scan images (0–45 μM) and 3D view of mouse tumor slice stained by **Probe 34**. (**2**) One-photon images of muscle tissue treated with **Probe 34** (20 μM) for 30 min. (**a**) Bright-field channel of the image. (**b**) The visible channel of the image. (**c**) NIR channel of the image. (**d**) The merged image of (**a**–**c**). (**e**–**f**) The one-photon confocal z-scan images (0–45 μM) and 3D view of mouse muscle slice stained by **Probe 34**. Reproduced with permission from ref. [86]. Copyright 2019 Elsevier.

In 2019, Xu et al. [86] designed and synthesized a dual-channel fluorescent probe, **Probe 34**, consisting of two fluorophores, cyanine (Cy) and dicyanomethylene-4H-pyran (DC), for selective recognition of GSH. As shown in Figure 42A, the probe can be used to detect GSH, Hcy, and Cys in different emission channels. After the addition of GSH, the probe showed significant fluorescence enhancement at both 520 nm and 810 nm at excitation wavelengths of 358 nm and 700 nm. According to the titration experiment of the probe with different concentrations of GSH in two different fluorescence emission channels, the detection limits of 24 nM and 32 nM were calculated, indicating that the probe is a highly sensitive fluorescence probe for the detection of GSH in vitro. The MTT test showed that the probe had good biocompatibility and very low toxicity. Confocal fluorescence imaging of U87 and L02 cells showed that the probe reacted with intracellular GSH to produce double fluorescence, which could simultaneously monitor endogenous GSH in both green and red channels. Fluorescence colocalization analysis showed that the probe had excellent mitochondrial localization ability and could distinguish between tumor and inflammatory sites in tumor-bearing mouse models (Figure 42B). In addition, the anatomy of the H22 tumor experiment demonstrated the potential of the probe as a fluorescent inducer for clinical use in nude mice.

**Figure 43:**
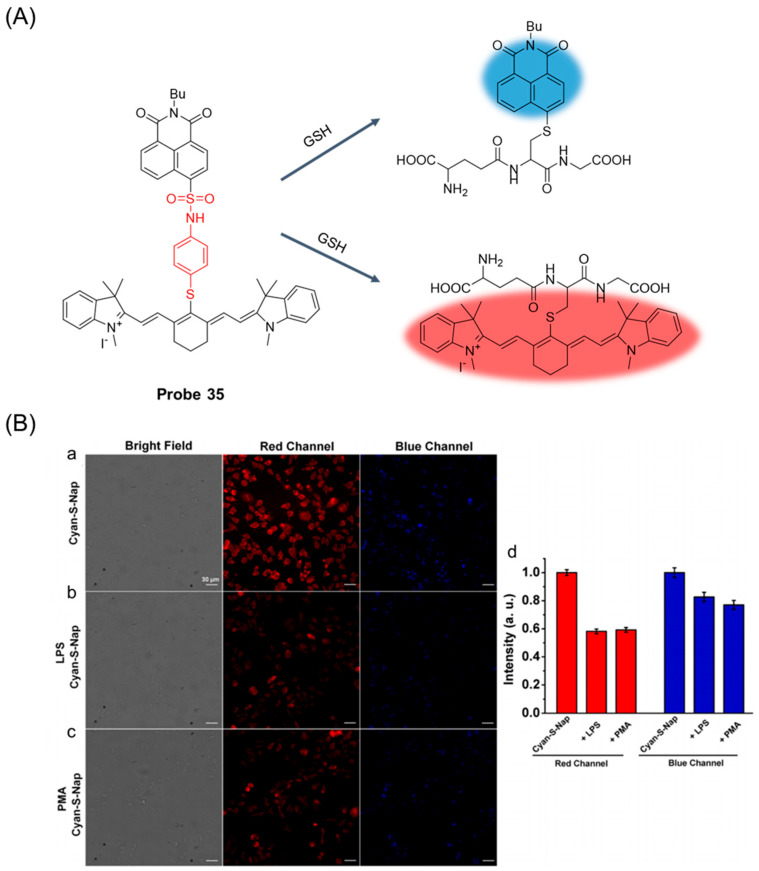
(**A**) Structure of **Probe 35** and its reaction with GSH. (**B**) Fluorescence imaging of **Cyan-S-Nap** (**Probe 35**) in RAW 264.7 cells. (**a**) Cells stained with **Cyan-S-Nap** (10 μM) alone for 30 min. Cells pretreated with LPS (1 μg/mL) (**b**) or PMA (1 μg/mL) (**c**) for 1 h and then stained with **Cyan-S-Nap** (10 μM) for 30 min. (**d**) Fluorescence quantification of a–d by ImageJ software (ImageJ). Blue channel: λ_ex_ = 350–404 nm, λ_em_ = 417–477 nm. Red channel: λ_ex_ = 617–645 nm, λ_em_ = 692–712 nm. Scale bar: 30 µm. Reproduced with permission from ref. [87]. Copyright 2023 Elsevier.

In 2023, Bao et al. [87] designed and synthesized a new double-reactive double-channel fluorescent probe, **Probe 35**, for selective detection of GSH. As shown in Figure 43A, the probe could simultaneously detect GSH in the near infrared and visible regions, using heptamethine cyanine and naphthalimide as fluorophores, respectively. After the addition of GSH, the fluorescence signal of the probe at 492 nm and 802 nm was significantly increased simultaneously. The probe had high selectivity and sensitivity to GSH, with detection limits of 5.68 μM and 0.36 μM in visible and near-infrared channels, respectively. In addition, cell imaging studies have shown that the probe has the ability to visualize intracellular GSH and to track ROS-induced changes in cellular GSH concentrations in biological systems (Figure 43B).

**Figure 44:**
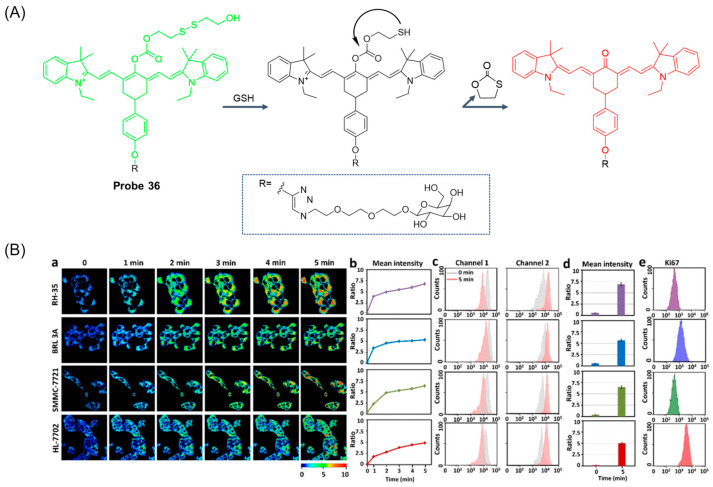
(**A**) Structure of **Probe 36** and its reaction with GSH. (**B**) Fluorescence imaging of the generation of endogenous GSH by **Probe 36** in living RH-35, BRL 3A, SMMC-7721, and HL-7702 cells. (**a**) Pseudocolored ratiometric images of endogenous GSH generation at different times: 0, 1, 2, 3, 4, and 5 min. (**b**) Average ratio intensities of **Probe 36** against time. (**c**) Flow cytometry results and (**d**) the corresponding mean ratio intensity at 0 and 5 min. (**e**) Histograms of Ki67 expression immunofluorescence response. The experiments were repeated three times, with the data shown as mean (±SD). The differences between 0 min and any other groups were analyzed via one-way ANOVA and Bonferroni post hoc test. The variance between the detected groups was analyzed via Student’s *t*-test. Reproduced with permission from ref. [88]. Copyright 2023 Royal Society of Chemistry.

In 2023, Han et al. [88] designed and synthesized a rate-type near-infrared fluorescence probe, **Probe 36**, for specific detection of GSH changes in living cells and mouse models of liver cancer. As illustrated in Figure 44A, the probe used heptamethine ketone cyanine as a fluorophore, bis (2-hydroxyethyl) disulfide as a fluorescence regulator, and D-galactose as a targeted part. After addition of GSH, the fluorescence of the probe at 783nm was decreased, and the fluorescence at 613nm was significantly enhanced. The fluorescence intensity ratio F_613nm_/F_783nm_ was positively correlated with the GSH concentration in the range of 0–10 mM, the actual detection limit was 2 μM, and the theoretical detection limit was 65 nM. Dynamic experiments showed that the probe reached a response plateau in 5 min after adding GSH (10 mM), which can be used as an effective tool for real-time bioimaging of intracellular GSH. In addition, probes have been shown to detect GSH production in live RH-35, BRL 3A, SMMC-7721, and HL-7702 cells (Figure 44B) as well as changes in endogenous GSH concentrations in live RH-35.

In this section, **Probes 33**–**36** are the focus of discussion for cyanine fluorophore, and Table 6 summarizes the selectivity, sensitivity, and applications of these probes. **Probes 33**, **34**, **35**, and **36** have high selectivity for GSH recognition, and **Probe 35** can recognize GSH simultaneously under different excitation wavelengths of two channels.

### 2.7. Fluorescent Probe Based on Benzothiazole

**Figure 45:**
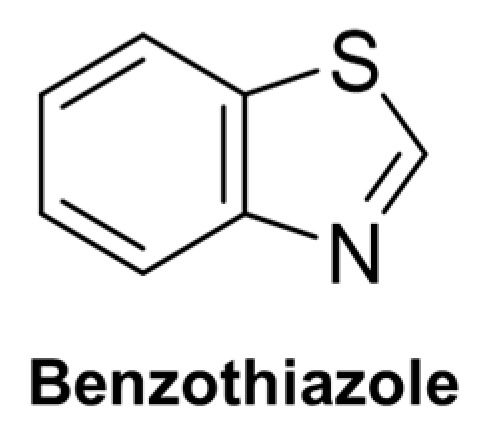
Structure of fluorophore benzothiazole.

The introduction of certain structures or functional groups on benzothiazole (Figure 45) and its derivatives containing benzene and thiazole rings can be used to construct benzothiazole fluorescent probes. Based on the mechanisms of photoinduced electron transfer (PET), excited state intramolecular proton transfer (ESIPT), intramolecular charge transfer (ICT), and aggregation-induced emission (AIE), the obtained fluorescence probes can interact specifically with the analyte to change its luminescence characteristics and realize the detection of the analyte [89]. Benzothiazole fluorescent probes show advantages such as a large Stokes shift, high quantum yield, and excellent color transformation and can be used for substance detection, harmful substance analysis, and cell imaging [90].

**Figure 46:**
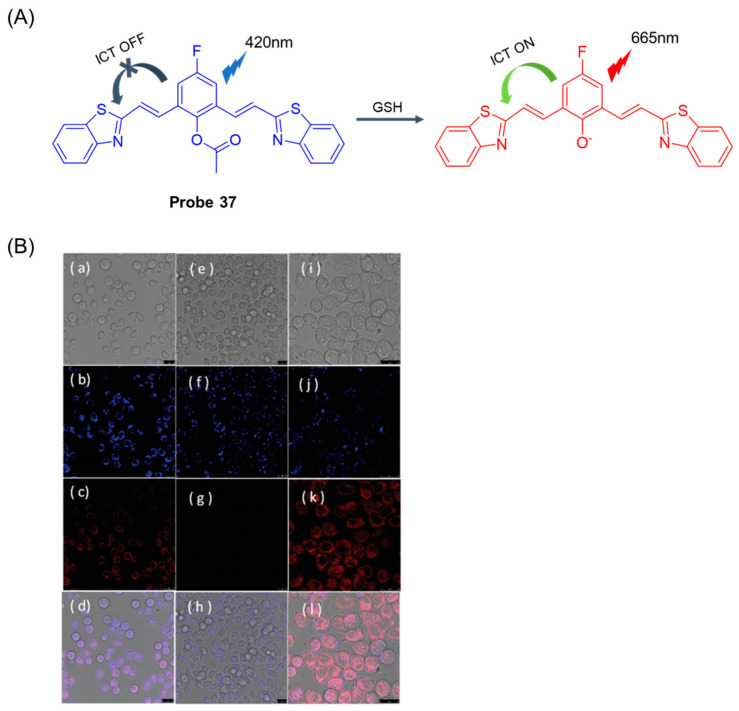
(**A**) Structure of **Probe 37** and its reaction with GSH. (**B**) Confocal fluorescence images of living HeLa cells incubated with **Probe 37** (20 μM) for 60 min at 37 °C: cells without treatment (**a**–**d**), cells with pre-treatment by NEM (1 mM) (**e**–**h**), and cells with pretreatment by NEM (1 mM) and the further addition of 200 μM GSH (**i**–**l**). (**a**,**e**,**i**) are the bright-field images; (**b**,**f**,**j**) and (**c**,**g**,**k**) are the fluorescence images at blue and red channels, respectively; (**d**,**h**,**l**) are the overlap of the fluorescence and bright-field images. Reproduced with permission from ref. [91]. Copyright 2019 Royal Society of Chemistry.

In 2019, Zhou et al. [91] developed a near-infrared ratio fluorescence probe, **Probe 37**, based on benzothiazole fluorophores. As shown in Figure 46A, in the composition of the probe, the active phenol group is masked by the acetyl group, which acts not only as a trigger for ICT fluorescence but also as a recognition site for GSH. In aqueous solution, the probe itself showed strong blue short-wavelength fluorescence emission at 426 nm and weak near-infrared fluorescence emission at 665 nm. When GSH was added, the near-infrared fluorescence was significantly enhanced, and the short-emission fluorescence was decreased. The selectivity experiments showed that the probe was highly selective to GSH and could distinguish the color changes before and after recognition. The fluorescence intensity ratio (I_665nm_/I_426nm_) was linearly correlated with the concentration of GSH from 0 to 100 μM, and the detection limit was 0.35 μM. In addition, the probe had low cytotoxicity and has been successfully used for rate-fluorescence bioimaging of GSH in living HeLa cells (Figure 46B).

**Figure 47:**
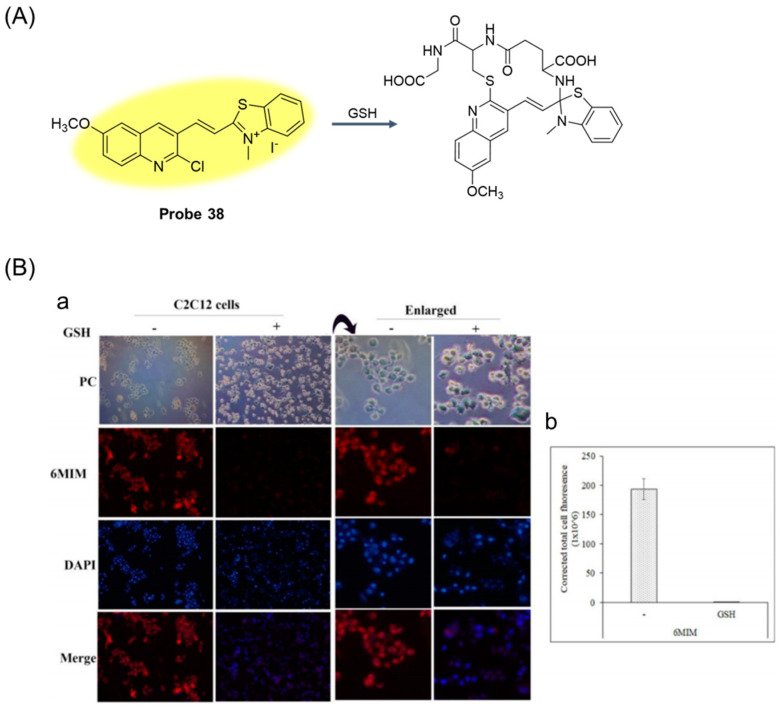
(**A**) Structure of **Probe 38** and its reaction with GSH. (**B**) GSH sensing in mammalian cells. Fluorescence microscope images of C2C12 cells treated with **6MIM** (**Probe 38**, 100 μM) in the presence or absence of GSH (500 μM), and the cells were also counter stained with nuclear stain DAPI (**a**). Cell images were obtained for **6MIM** and DAPI using an excitation filter BP545–580 and BP330–385, respectively. Magnification 10×. Quantification of fluorescence signal in these cells is shown in the graph (**b**). Error bar indicates +/− standard error of the mean (SEM). Reproduced with permission from ref. [92]. Copyright 2020 Elsevier.

In 2020, Mani et al. [92] designed and synthesized a quinoline semi-cyanine fluorescent probe, **Probe 38**, with strong ICT properties for selective detection of GSH. As shown in Figure 47A, after adding GSH, the color of the solution changed from yellow to colorless, and the fluorescence quantum yield decreased from 15% to 1.3%. As the concentration of GSH increased, the emission peak decreased steadily to 522 nm and a new emission peak appeared at 428 nm. The fluorescence intensity and GSH concentration increased linearly in the range of 30 μM–75 μM, and the detection limit was 100 nM. Moreover, the probe had the advantages of a simple preparation method, good light stability, and good performance. Cell experiments have shown that the probe has low cytotoxicity and can be used as a potential tool to detect GSH in living cells (Figure 47B). In addition, the probe was used in conjunction with a smartphone RGB color value application, enabling real-time online analysis of GSH with a minimum detection limit of 120 nM.

In this section, **Probes 37** and **38** are the focus of discussion for benzothiazole fluorophore, and Table 7 summarizes the selectivity, sensitivity, and applications of these probes. **Probes 37** and **38** have high selectivity and sensitivity for GSH recognition, among which **Probe 38** can be used in combination with smartphone RGB color value applications.

### 2.8. Other GSH Fluorescent Probes

**Figure 48:**
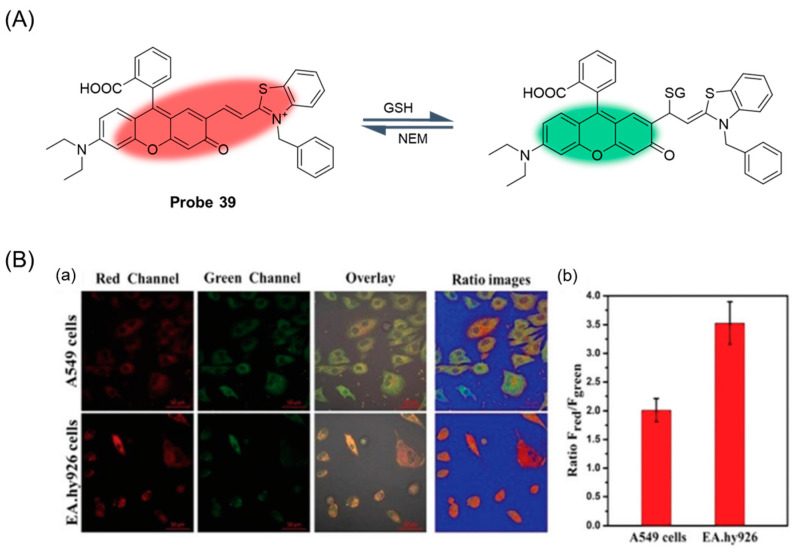
(**A**) Structure of **Probe 39** and its reaction with GSH. (**B**) (**a**) Confocal fluorescence images of A549 cells and EA.hy926 cells treated with **Probe 39** (4 µM) for 20 min. (**b**) F_red_/F_green_ ratios based on the average fluorescence intensity of the imaging results in (**a**). Scale bar: 50 μm. Reproduced with permission from ref. [93]. Copyright 2019 Royal Society of Chemistry.

In 2019, Ren et al. [93] developed a reversible fluorescent probe, **Probe 39**, (Figure 48A) based on rhodol-hemocyanin fluorescent dye to detect changes in intracellular GSH levels. After the addition of GSH, the maximum emission peak of the probe at 688 nm gradually decreased, while a new emission peak appeared at 560 nm. The fluorescence intensity ratio I_560nm_/I_688nm_ showed a good linear relationship with GSH concentration in the range of 0–7 mM, and the detection limit was 9.43 μM. Moreover, the probe was able to respond quickly with GSH within 1 min, and the half-life (t_1/2_) was calculated to be 89 ms. Moreover, the probe could detect GSH under physiological pH conditions and had high selectivity and good photostability. In addition, the probe had low cytotoxicity and has been successfully applied to fluorescence imaging of GSH concentration fluctuations in living cells (Figure 48B).

**Figure 49:**
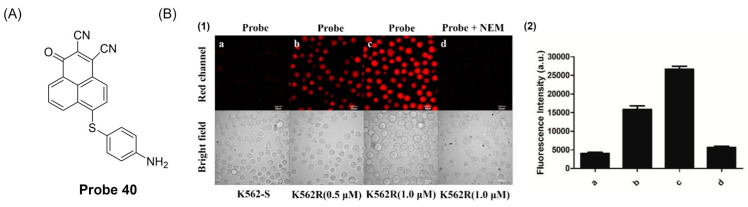
(**A**) Structure of **Probe 40**. (**B**) (**1**) Fluorescence imaging in K562S and K562R cells. K562S cells (**a**), K562R (0.5 μM) cells (**b**), and K562R (1.0 μM) cells (**c**) incubated with **Probe 40** (5 μM) in PBS (10 mM, pH 7.4, containing 1% DMSO) for 30 min at 37 °C. (**d**) K562R (1.0 μM) cells were pretreated with NEM (500 μM) for 30 min then further incubated with **Probe 40** (5 μM) for 30 min. Emission was collected at 600–650 nm under excitation with a 488 nm laser. Scale bar = 20 μm. Fluorescence imaging of all groups were performed under the same experimental conditions. (**2**) Quantitative image analysis of the average total fluorescence of the (a–d) group was determined from the analysis of 20 cells in each sample image. Reproduced with permission from ref. [94]. Copyright 2019 Royal Society of Chemistry.

In 2019, Zhang et al. [94] developed a fluorescence probe, **Probe 40**, (Figure 49A) using OPD as a fluorophore based on the S_N_Ar mechanism. When GSH was added, the fluorescence emission was significantly enhanced at 606 nm and showed high selectivity. The probe was stable under physiological pH conditions and showed significant fluorescence response to GSH when the pH value was 7.0–8.0. The fluorescence intensity after recognition increased linearly with the increase in GSH concentration in the range of 0–10 μM, and the detection limit was 2.3 × 10^−8^ M. In addition, the MTT test proved that the probe had low cytotoxicity. The probe has been successfully applied to fluorescence imaging of GSH in living cells and organisms, and for the first time it measured GSH levels in different imatinib-resistant K562 tumor cells (Figure 49B).

**Figure 50:**
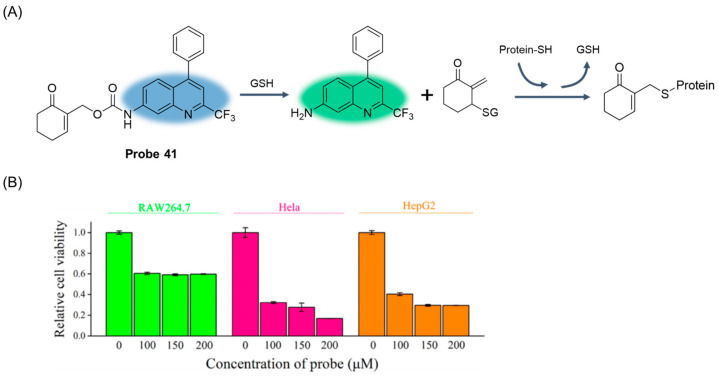
(**A**) Structure of **Probe 41** and its reaction with GSH. (**B**) Cytotoxicity assays of GT-GSH with different concentrations (0–200 μM) for RAW264.7, HeLa, and HepG2 cells. Reproduced with permission from ref. [95]. Copyright 2021 American Chemical Society.

In 2021, Rong et al. [95] designed and synthesized a fluorescence probe, **Probe 41**, targeting GSH in the Golgi apparatus, with 4-CF3-7-aminoquinoline as the fluorescent body. After the addition of GSH, the fluorescence intensity of the probe at 425 nm decreased and a new absorption peak appeared at 510 nm. The fluorescence intensity ratio I_510nm_/I_425nm_ showed a significant linear relationship with the concentration of GSH in the range of 0–75 μM, and the detection limit was 0.49 μM. As illustrated in Figure 50A, after the probe reacted with GSH, the ester group was broken and the active molecule and 4-CF3-7-aminoquinoline fluorescence were released. The active molecule covalently binds to the mercaptan on the Golgi protein, causing the abnormality of the Golgi apparatus, thus playing a role in killing cancer cells. In addition, the probe has been successfully applied to track changes in GSH concentration levels in the Golgi apparatus by ratio fluorescence imaging. More importantly, the probe is selectively toxic to cancer cells (Figure 50B) and is expected to be used as a new cancer treatment drug in the comprehensive course of cancer treatment.

**Figure 51:**
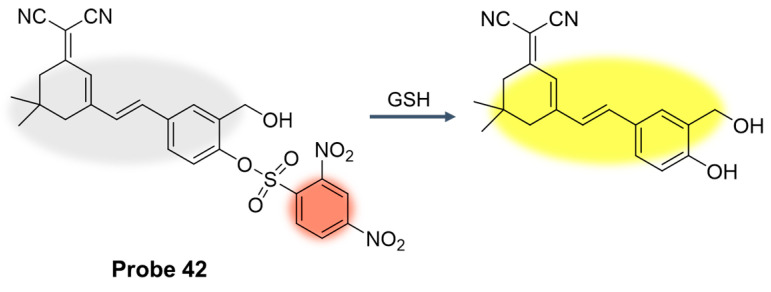
Structure of **Probe 42** and its reaction with GSH. Reproduced with permission from ref. [96]. Copyright 2022 American Chemical Society.

In 2022, Yuan et al. [96] prepared four kinds of fluorescent probes by adjusting the functional group at the ortho position of the dicyanoisophorone core. The results of fluorescence response and molecular simulation showed that **Probe 42** showed the strongest binding affinity. As shown in Figure 51, the probe used dinitrobenzene sulfonic acid as a recognition site and fluorescence quencher. The probe showed a dual-channel spectral response to human serum albumin (HSA) and GSH. After the addition of HSA, a fluorescence emission at 660 nm was observed under excitation of 500 nm, while the presence of GSH did not cause interference. The probe showed a good linear relationship with HSA in the range of 0.5–18 μM, and the detection limit was 35 nm. However, by switching the excitation wavelength to 400 nm, the probe reacted with GSH with a yellow fluorescence emission at 575 nm. The probe showed a good linear relationship with GSH concentration in the range of 5–60 μM, and the detection limit was 4.65 μM. In addition, the cell imaging results showed that the probe had low cytotoxicity and is expected to be applied to monitor the changes in HSA and endogenous/exogenous GSH in HepG2 cells.

**Figure 52:**
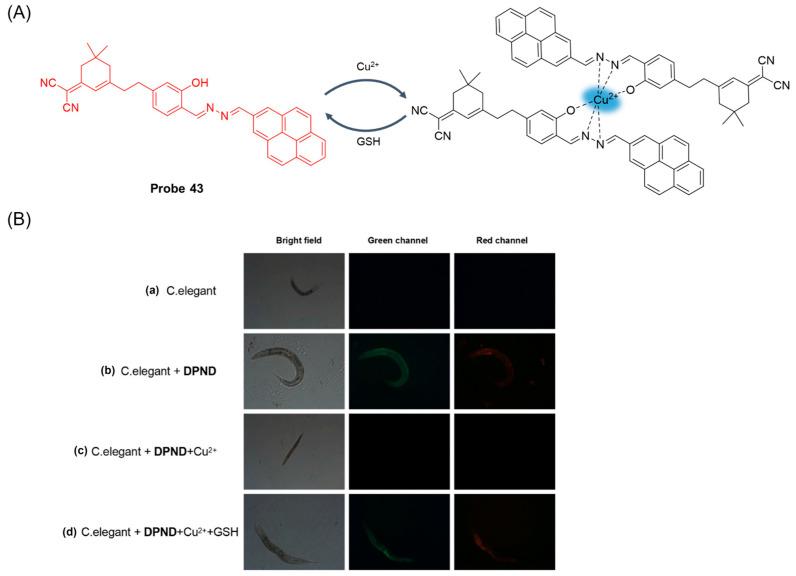
(**A**) Structure of **Probe 43** and its reaction with GSH. (**B**) Fluorescence microscope imaging of *C. elegans*. (**a**) Fluorescence images of *C. elegans*. (**b**) Fluorescence images of *C. elegans* treated with **DPND** (**Probe 43**, 10 μM, DMSO/H_2_O = 2:3, *v*/*v*). (**c**) Fluorescence images of **DPND** (10 μM)-loaded *C. elegans* treated with Cu^2+^ (30 μM). (**d**) Fluorescence images of **DPND** (10 μM) + Cu^2+^ (5 μM)-loaded *C. elegans* treated with GSH (100 μM). From left to right is bright-field, green channel, red channel. Reproduced with permission from ref. [97]. Copyright 2023 Elsevier.

In 2023, Yao et al. [97] designed and synthesized a novel AIE fluorescent probe, **Probe 43**, for GSH detection. As illustrated in Figure 52A, the probe first underwent the “off” property of coordination with the metal cation Cu^2+^, which reduced the fluorescence intensity of the probe itself to 1/10th of the original. After adding GSH, 43-Cu^2+^ highly selectively recognized GSH and produced a fluorescent “on” property. The fluorescence intensity was linearly proportional to GSH concentration in the range of 0.0–80 μM, and the detection limit was 1.17 μM. The response time of the probe and Cu^2+^ was 180 s, and the response time of probe 43-Cu^2+^ and GSH was 360 s, both of which had fast response speed. In addition, the probe showed a reversible “off–on” fluorescence response and good stability during the alternations of Cu^2+^ and GSH. The fluorescence intensity of the probe did not change in the range of pH 5.0–8.0, indicating that it was suitable for physiological conditions. Most importantly, the improved probe showed extremely low cytotoxicity and could be used to determine GSH changes in mouse, *C. elegans* (Figure 52B), and Hela cells.

In this section, **Probes 39**–**43** are the focus of discussion for other fluorophores. Other fluorophores include Rhodro-hemocyanin fluorescent dye, 1-oxo-1H-phenalene-2,3-dicarbonitrile (OPD), 4-CF3-7-aminoquinoline, and dinitro-benzene sulfonic acid, and Table 8 summarizes the selectivity, sensitivity, and applications of these probes. **Probes 39**, **40**, **41**, and **43** have high selectivity for GSH recognition, and **probe 39** can react with GSH quickly and reversibly in the presence of NEM. **Probe 42** belongs to the dual-channel identification of GSH and other substances, and **probe 43** is a probe with AIE characteristics.

## 3. Conclusions

GSH plays an important role in living organisms, and abnormal levels of GSH are thought to be associated with the occurrence of many diseases. Therefore, the development of fluorescent probes that can be used to monitor GSH concentration levels in organisms is very important, which can help in the diagnosis and treatment of diseases. According to the chemical reaction properties of GSH, the GSH selective fluorescent probe was constructed using a variety of recognition mechanisms, such as Michael addition, aromatic nucleophilic substitution (S_N_Ar), intramolecular charge transfer (ICT), photoinduced electron transfer (PET), fluorescence resonance energy transfer (FRET), and aggregation-induced emission (AIE). In this paper, we reviewed the sensitive and selective detection of GSH by fluorescent probes developed within the past five years, classified them according to fluorophore types, and systematically discussed the synthesis methods, detection mechanisms, photophysical properties, and bioimaging applications of the probes. However, in the process of developing GSH fluorescent probes, there are still some problems that need to be further studied. These challenges can be summarized as follows: (1) the need to develop highly selective and specific GSH fluorescent probes; (2) new fluorophores requiring low cost and high fluorescence quantum yield; (3) more reaction mechanisms need to be explored to improve the response speed and sensitivity of the probe; and (4) the actual clinical application of GSH fluorescent probes still needs further research. In short, we hope that this review will be helpful to researchers in related fields and inspire them to develop next-generation GSH fluorescent probes. We believe that GSH fluorescent probes can eventually be widely used in the diagnosis and treatment of diseases and as a powerful tool for further research in the fields of chemistry, biology, food, and medicine.

## Figures and Tables

**Table 1:** Summary of the relevant parameters of GSH probes based on BODIPY fluorophore.

Number	Original Name	Merits	λ_ex_ (nm)	λ_em_ (nm)	LOD	Applications	Reference
**Probe 1**	1	High selectivity	500	544	/ *	HeLa	[43]
**Probe 2**	1	Dual-channel	538	558	5.6 × 10^−8^ M	Raw264.7	[44]
**Probe 3**	ST-BODIPY	High selectivity, High sensitivity	670	719	25.46 nM	MCF-7	[45]
**Probe 4**	BSR1	High selectivity, High sensitivity	600	663	83 nM	NCI-H1975, HepG2, Mouse	[46]
**Probe 5**	1	Ratiometric, Reversible	F_488_/F_543_	F_544_/F_608_	/ *	HeLa, A549-DDP, A549	[47]
**Probe 6**	^α^BD-GSH	Quick response, Reversible	594	609	3.4 μM	A549, HeLa, 293T	[48]
**Probe 7**	1	High selectivity, High sensitivity	532	595	2.9 × 10^−8^ M	HUVECs, Zebrafish	[49]
**Probe 8**	DiOH-BDP	High selectivity, Reversible	/ *	/ *	7.2 μM	Raw264.7, AML-12	[50]
**Probe 9**	1	High selectivity, Three channels	540	558	2.3 × 10^−7^ M	H9C2, Mouse	[51]

* “/” Means not mentioned in the literature.

**Table 2:** Summary of the relevant parameters of GSH probes based on 1,8-Naphthalimide fluorophore.

Number	Original Name	Merits	λ_ex_ (nm)	λ_em_ (nm)	LOD	Applications	Reference
**Probe 10**	GP5	High selectivity	405	496	0.11 μM	SH-SY5Y, Primary cortical neuronal cells	[54]
**Probe 11**	TAT-probe	Dual-channel, Ratiometric	545	585	5.15 μM	HeLa	[55]
**Probe 12**	1	High selectivity, Two-photon	350	495	9.3068 × 10^−3^ M	Primary cortical neuronal cells, Section of rat hippocampus	[56]
**Probe 13**	ER-G	High selectivity, High sensitivity	405	523	0.12 μM	HeLa	[57]
**Probe 14**	R13	High selectivity	390	498	/ *	HeLa, HepG2, Mouse live, Mouse Parkinson’s disease model	[58]

* “/” Means not mentioned in the literature.

**Table 3:** Summary of the relevant parameters of GSH probes based on coumarin fluorophore.

Number	Original Name	Merits	λ_ex_ (nm)	λ_em_ (nm)	LOD	Applications	Reference
**Probe 15**	QG-1	High selectivity, Ratiometric, Reversible, Quick response	450	F_488_/F_560_	/ *	HeLa	[61]
**Probe 16**	Cou-Br	High selectivity, High sensitivity, Two-photon	460	520	90 μM	FaDu, A549, HepG2, OVCRA-3, SGC-7901	[62]
**Probe 17**	CBF3	High selectivity, High sensitivity	455	495	9.2 nM	HepG2, *C. elegans*	[63]
**Probe 18**	1	High selectivity, High sensitivity	370	471	5.5 nM	BEL-7402, Zebrafish, Raw264.7	[64]
**Probe 19**	RP-2	High selectivity, Ratiometric, Reversible, Quick response	F_420_/_530_	F_505_/F_587_	/ *	HeLa	[65]
**Probe 20**	GScp	High selectivity, Reversible, Ratiometric	F_350_/F_410_	F_460_/F_510_	0.245 μM	A549, HepG2, HeLa, SW-480, LO2, BEAS-2B	[66]
**Probe 21**	DCM-Cou-SePh	Dual-channel, High sensitivity	430	530	0.12 μM	MGC-803, Raw264.7	[67]
**Probe 22**	GSH547	High selectivity, High sensitivity, Ratiometric	F_440_/F_560_	F_547_/F_650_	0.16 μM	HeLa, Liver tissue	[68]
**Probe 23**	QG-S	Reversible, Good stability	405	486	4.5 μM	HeLa	[69]
**Probe 24**	FC-NBD	Dual-channel, High sensitivity	470	660	0.199 μM	HeLa, Zebrafish	[70]
**Probe 25**	rCP-NN-βCD	Reversible, High sensitivity, Ratiometric	F_409_/F_506_	F_470_/F_566_	148 nM	HeLa	[71]
**Probe 26**	SWJT-14	Good stability	490	553	0.92 μM	HeLa	[72]

* “/” Means not mentioned in the literature.

**Table 4:** Summary of the relevant parameters of GSH probes based on xanthene fluorophore.

Number	Original Name	Merits	λ_ex_ (nm)	λ_em_ (nm)	LOD	Applications	Reference
**Probe 27**	ADS	High selectivity	550	630	13.1 μM	HeLa	[75]
**Probe 28**	EQR-S	High selectivity, High sensitivity, Quick response, Reversible	585	728	69 nM	HL-7702, HepG2	[76]
**Probe 29**	GeP	High selectivity, High sensitivity, Reversible, Quick response	595	644	0.07 μM	HeLa	[77]

**Table 5:** Summary of the relevant parameters of GSH probes based on rhodamine fluorophore.

Number	Original Name	Merits	λ_ex_ (nm)	λ_em_ (nm)	LOD	Applications	Reference
**Probe 30**	RBA	High selectivity	560	585	1.0 μM	MCF-7	[80]
**Probe 31**	Rho-GSH	High selectivity, Quick response	510	557	49.6 μM	HepG2, Zebrafish, LO2	[81]
**Probe 32**	F1	High selectivity	450	530	37 μM	RKO, DLD-1, HCT116, CaCO_2_, Mgc-803, NCIN87, CP HO48, FHS 74Int, MC-38	[82]

**Table 6:** Summary of the relevant parameters of GSH probes based on cyanine fluorophore.

Number	Original Name	Merits	λ_ex_ (nm)	λ_em_ (nm)	LOD	Applications	Reference
**Probe 33**	Cy-GST	High selectivity, High sensitivity	730	810	10 ng/mL	IMR-90, RLE-6TN, Mouse	[85]
**Probe 34**	Cy-DC	High selectivity, High sensitivity, Dual-channel	358/700	520/810	24/32 nM	L02, U87MG, Mouse	[86]
**Probe 35**	Cyan-S-Nap	Dual-channel, High selectivity	380/492	720/810	5.68/0.36 μM	HeLa, HepG2, Raw264.7	[87]
**Probe 36**	CyO-Disu	Ratiometric, High selectivity, High sensitivity	F_535_/F_710_	F_613_/F_783_	65 nM	RH-35, BRL 3A, SMMC-7721, HL-7702	[88]

**Table 7:** Summary of the relevant parameters of GSH probes based on benzothiazole fluorophore.

Number	Original Name	Merits	λ_ex_ (nm)	λ_em_ (nm)	LOD	Applications	Reference
**Probe 37**	HBT-GSH	Ratiometric, High selectivity	350	F_665_/F_426_	0.35 μM	HeLa	[91]
**Probe 38**	6MIM	High selectivity, High sensitivity, Smartphone integration	380	F_428_/F_522_	100 nM	C2C12	[92]

**Table 8:** Summary of the relevant parameters of GSH probes based on other fluorophores.

Number	Original Name	Merits	λ_ex_ (nm)	λ_em_ (nm)	LOD	Applications	Reference
**Probe 39**	RdH	Ratiometric, High selectivity, Quick response, Reversible	F_510_/F_610_	F_560_/F_688_	9.43 μM	A549, EA.hy926	[93]
**Probe 40**	O-NH_2_	High selectivity, High sensitivity	474	606	2.3 × 10^−8^ M	HeLa, K562S, K562R, D. magna, zebrafish	[94]
**Probe 41**	GT-GSH	Ratiometric, High selectivity	320	F_510_/F_425_	0.49 μM	HeLa, Raw264.7, HepG2	[95]
**Probe 42**	M-OH-SO_3_	Dual-channel	400	575	4.65 μM	HepG2	[96]
**Probe 43**	DPND	AIE, High selectivity	/ *	F_624_/F_594_	1.17 μM	*C. elegans*, HeLa, Mouse	[97]

* “/” Means not mentioned in the literature.

## Data Availability

No new data were created or analyzed in this study. Data sharing is not applicable to this article.
